# Detection and Organ-Specific Ablation of Neuroendocrine Cells by *Synaptophysin* Locus-Based BAC Cassette in Transgenic Mice

**DOI:** 10.1371/journal.pone.0060905

**Published:** 2013-04-22

**Authors:** Chieh-Yang Cheng, Zongxiang Zhou, Alexander Yu. Nikitin

**Affiliations:** Department of Biomedical Sciences, Cornell University, Ithaca, New York, United States of America; Roswell Park Cancer Institute, United States of America

## Abstract

The role of cells of the diffuse neuroendocrine system in development and maintenance of individual organs and tissues remains poorly understood. Here we identify a regulatory region sufficient for accurate *in vivo* expression of synaptophysin (SYP), a common marker of neuroendocrine differentiation, and report generation of Tg(*Syp-EGFP^loxP^-DTA)147^Ayn^* (*SypELDTA*) mice suitable for flexible organ-specific ablation of neuroendocrine cells. These mice express EGFP and diphtheria toxin fragment A (DTA) in SYP positive cells before and after Cre-*loxP* mediated recombination, respectively. As a proof of principle, we have crossed *SypELDTA* mice with *EIIA-Cre* and *PB-Cre4* mice. *EIIA-Cre* mice express Cre recombinase in a broad range of tissues, while *PB-Cre4* mice specifically express Cre recombinase in the prostate epithelium. Double transgenic *EIIA-Cre; SypELDTA* embryos exhibited massive cell death in SYP positive cells. At the same time, *PB-Cre4; SypELDTA* mice showed a substantial decrease in the number of neuroendocrine cells and associated prostate hypotrophy. As no increase in cell death and/or Cre-*loxP* mediated recombination was observed in non-neuroendocrine epithelium cells, these results suggest that neuroendocrine cells play an important role in prostate development. High cell type specificity of *Syp* locus-based cassette and versatility of generated mouse model should assure applicability of these resources to studies of neuroendocrine cell functions in various tissues and organs.

## Introduction

Neuroendocrine (NE) cells have both neuronal and endocrine phenotypes [1]. The diffuse neuroendocrine system (DNES) is composed of NE cells scattered throughout the entire body either as single cells or clusters, such as solitary pulmonary NE cells (PNECs) and neuroepithelial bodies (NEBs) [2], the islets of Langerhans in the pancreas [3], [4], gastrointestinal NE cells [5], [6], dermal NE cells (so-called Merkel cells) [7], adrenal medullary NE cell [8]–[10], and prostate NE cells [11]. PNECs are implicated in regulation of lung maturation and growth, function as oxygen-sensing chemoreceptors and are likely important for lung stem cell niches [2]. Gastrointestinal NE cells are known to control gastrointestinal secretion, motility, growth, immune cell function and food intake [5]. Though there has been progress in understanding the function of NE cells, the physiological role of NE cells in most other organs is not well understood.

Cells with NE differentiation are also present in many cancer types, with their representation ranging from being the major component in small cell carcinomas of the lung [2] and prostate [12], as well as NE tumors of gastrointestinal tract [13], to more limited quantity in other cancers, such as adenocarcinomas of the lung [2] and prostate [12]. Unfortunately, the cell of origin of neoplastic NE cells and their contribution to cancer progression remain insufficiently elucidated [1], [2], [12], [14].

NE cells are detected by a number of markers, such as chromogranin A (CgA) [15], neuron-specific enolase (NSE) [16], neural cell adhesion molecules (NCAMs, so-called CD56) [17], calcitonin gene-related peptide (CGRP) [18] and SYP [19]. However, the use of NSE [20]–[22] or CD56 [23], [24] is limited because of their poor specificity and/or sensitivity. CgA reactivity is strongly dependent on the number of neurosecretory vesicles per cell and is frequently lost in neoplastic NE cells [25], while only subset of NE cells expresses CGRP [26]. In contrast, SYP is expressed in a broad-spectrum of normal and neoplastic NE and neural cells [19], [27].

SYP is a major integral membrane protein of small synaptic vesicles and belongs to a family of proteins that includes synaptogyrin (SYG) and synaptoporin [28]. It has been reported that in cell culture transfection experiments the 1.2 kb upstream region of rat *Syp* promoter is insufficient to confer cell type specific expression [29]. It has also been suggested that NE cell specific silencer elements lay within the 2.6 kb upstream fragment of *Syp* [29]. At the same time, other cell culture studies have reported that neuron-restrictive silencer element *(NRSE),* a binding site for RE-1 silencing transcription factor (REST), a.k.a. neuron-restrictive silencer factor (NRSF), is located within the first intron of *Syp* gene [30]. However, the regulatory region sufficient for accurate *in vivo* expression of SYP remains unknown, thereby preventing development of genetic constructs allowing *Syp*-specific gene expression.

Since SYP is among the most reliable markers for NE cells, we generated mice with *Syp* locus-based Bacterial Artificial Chromosome (BAC) cassettes. We show that in combination with the preserved *NRSE* in the first intron, only the 121 kb upstream and 36 kb downstream regions, but not the 3 kb upstream region, allow for accurate expression of reporter gene in SYP expressing cells in the mouse. We also show that SYP positive cells can be accurately ablated in either the embryo or in the postnatal adult prostate after induction of DTA expression [31] by Cre-*loxP* mediated recombination in crosses of *SypELDTA* mice with *EIIA-Cre* [32] or *PB-Cre4* [33] mice, respectively. The *Syp* containing BAC cassette and generated mice should provide useful tools for studies of NE cell biological roles in development and maintenance of various tissues and organs.

## Materials and Methods

### Bioinformatics Analyses

Analysis of sequence and species comparisons were performed by using the University of California Santa Cruz Genome Browser (UCSC, http://genome.ucsc.edu/).

### Generation of *SypELDTA* Mice

A BAC clone containing approximately 121 kb and 36 kb of 5' and 3' DNA flanking the *Syp* locus was modified by insertion of a *loxP-EGFP-Neo cassette-Stop-loxP-DTA-bpA* cassette to replace the sequence spanning intron 1 downstream of *NRSE* to exon 7 of *Syp* locus by homologous recombination. The BAC constructs were microinjected into male pronuclei of fertilized oocytes from FVB/N mice to generate the *SypELDTA* mice. *EIIA-Cre* (FVB/N-Tg(EIIa-cre)C5379Lmgd/J) transgenic mice (The Jackson Laboratory, Bar Harbor, ME, stock number #003314) [32], *Rosa26Stop^loxP^LacZ* (B6;129S4-*Gt(ROSA)26Sor^tm1Sor^*/J) reporter mice (The Jackson Laboratory, stock number #003309) [34], and *ARR_2_PB-Cre* transgenic male mice on FVB/N (*PB-Cre4*) [33] were described previously. Details about generation of the targeting construct and BAC recombineering are described in the Materials and Methods S1.

### Ethics Statement

This study was carried out in strict accordance with the recommendations of the Guide for the Care and Use of Laboratory Animals of the National Institutes of Health. The protocol was approved by the Institutional Laboratory Animal Use and Care Committee at Cornell University (Permit Number: 2000–0116). All efforts were made to minimize animal suffering.

### Histotechnology

Mice euthanized according to schedule were subjected to cardiac perfusion by phosphate-buffered 4% paraformaldehyde. After digital camera photography during necropsy, collected tissues were processed for embedding in paraffin. Histological evaluations were done on 4 µm-thick sections stained with hematoxylin (Mayer's haemalum) and eosin. Transverse sections of the whole prostate were scanned by ScanScope (Aperio Technologies, Vista, CA) with 40×objective followed by lossless compression and assessment of all alterations in identical anatomic regions.

### Immunohistochemical Analyses

Immunoperoxidase staining of paraffin sections of paraformaldehyde-fixed tissue was performed by a modified avidin-biotin-peroxidase (ABC) technique [35]. Antigen retrieval was done by boiling the slides in 10 mM citric buffer (pH 6.0) for 10 minutes. The primary antibodies to cytokeratin-8 (CK8), cytokeratin-5 (CK5), SYP and cleaved Caspase-3 were incubated with deparaffinized sections at 4°C overnight. After incubation with methanol (Fisher Scientific, Bohemia, NY, #A454-4) containing 0.3% hydrogen peroxide (Sigma, St. Louis, MO, #H1009), sections were subsequently incubated with biotinylated secondary antibody for 30 minutes at room temperature and subsequently detected with the ABC Elite kit (Vector Laboratories, Burlingame, CA, #PK-6100) and 3,3-diaminobenzidine (DAB; Sigma, #D4418) as substrate. Hematoxylin was used as the counterstain in immunoperoxidase stainings. Double immunofluorescence staining was performed by incubation of SYP and EGFP, or β-galactosidase primary antibody at 4°C overnight, followed by Alexa-Fluor 594-conjugated and Alexa-Fluor 488- conjugated secondary antibodies. To stain cell nuclei, sections were incubated with a 10 µg/ml solution of 4',6-diamidino-2-phenylindole (DAPI; Sigma, #D9542) for 3 min. Antibody sources and dilutions are listed in the Materials and Methods S1.

### Morphometric quantitative analyses

Five digital images of serial sections scanned by ScanScope with 40× objective were captured for each slide and transferred to Image J for manual counting of all epithelial cells (at least 1,000 cells) and SYP positive cells among them. The sizes of prostate lobes were determined by measuring the distance from the edge of each lobe to the urethra. The size of each prostatic duct was determined by measuring its diameter.

### Western Blot Analyses

PCN1, PCN2, and PCN3 cell lysates were prepared using RIPA buffer (Tris-HCl 50 mM, pH 7.4; Nonidet P−40 1%; Na-deoxicholate 0.25%; NaCl 150 mM; EDTA 1 mM; PMSF 1 mM; Aprotinin, leupeptin, pepstatin: 1 µg/mL each; Na_3_VO_4_ 1 mM; NaF 1 mM), separated by 12% SDS-PAGE and transferred to PVDF membrane (Millipore). The membrane was incubated overnight at 4°C with antibodies to detect SYP (Dako, CA, #A0010, 1∶100), followed by incubation for 1 hour at room temperature with horseradish peroxidase-conjugated secondary antibodies (Santa Cruz Biotechnology, CA, #sc-2301, 1∶2000) and developed using chemiluminescent substrate (Thermo scientific, Rockford, IL, #34077).

### Statistical Analyses

Statistical analyses were performed with InStat 3.10 and Prism 5.01 software. (GraphPad, Inc., San Diego, CA). Two-tailed unpaired *t*-test was used in all calculations.

## Results

### Genomic Structure of *Syp* Locus and BAC Engineering for the Generation of *SypELDTA* Constructs

**Figure 1:**
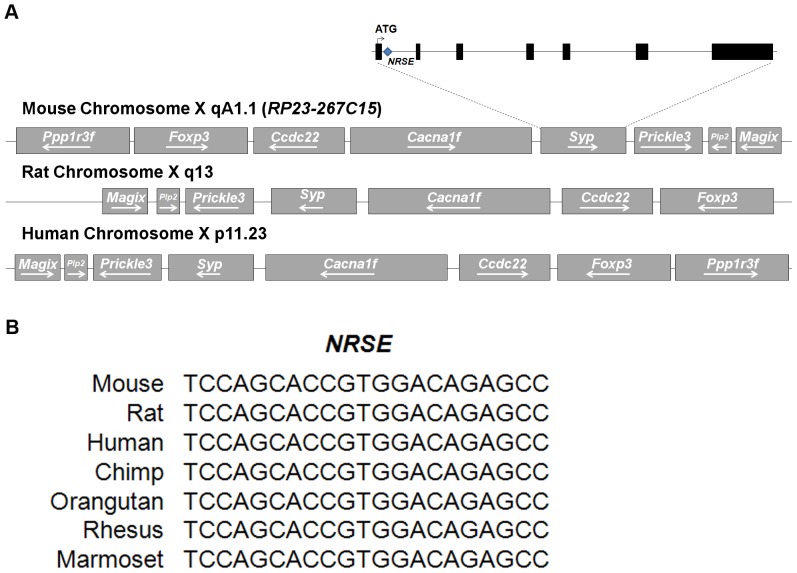
Genomic structure of the *Syp* gene. (A) Location of the *Syp* locus on mouse, rat, and human chromosome X. Mouse *Syp* contains 7 exons (black boxes). The translation initiation codon, ATG, is located in the first exon. The *NRSE* is located within the first intron of *Syp*. (B) Sequence comparison of the *NRSE* derived from *Syp* across species.

**Figure 2:**
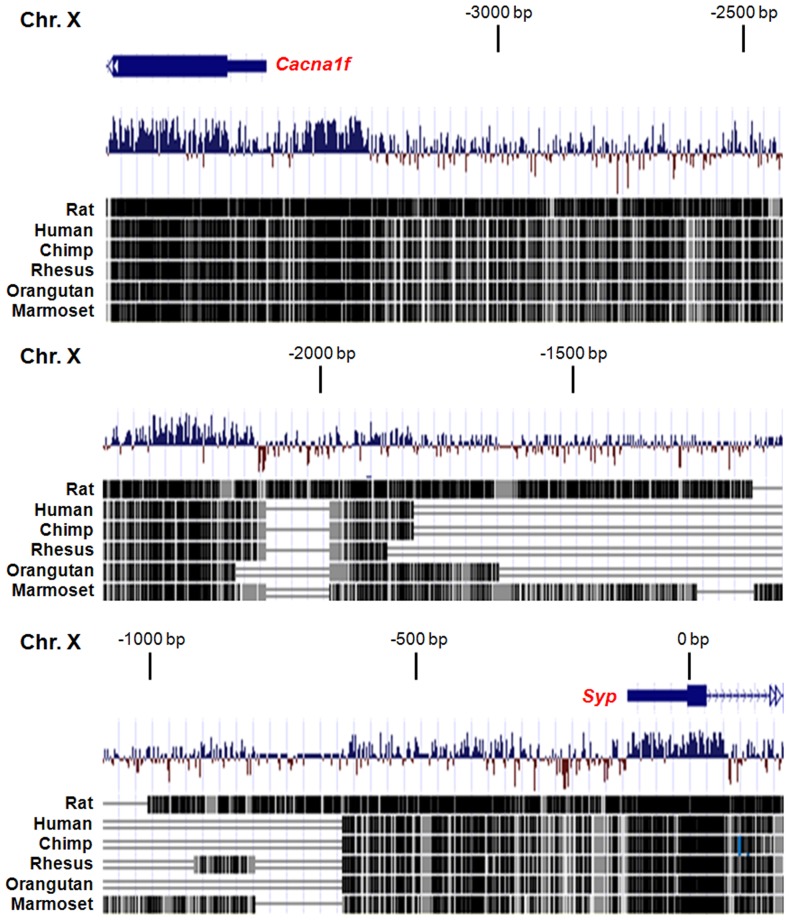
Sequence comparison of upstream region of *Syp* across species. Black lines and boxes represent highly conserved areas of the *Syp* upstream region. The position of upstream sequence is relative to the *Syp* transcription start site (0 bp). The assembly dates of the upstream regions are July 2007 (*Mus musculus*), November 2004 (*Rattus norvegicus*), February 2009 (*Homo sapiens*), October 2010 (*Pan troglodytes*), January 2006 (*Macaca mulatta*), July 2007 (*Pongo pygmaeus abelii*), and March 2009 (*Callithrix jacchus*).

To identify the region containing all transcriptional *cis*-elements sufficient for accurate SYP expression *in vivo*, we have analyzed the genomic *Syp* locus by using the UCSC Genome Browser. The *Syp* locus is located on mouse chromosome×and contains 7 exons and 6 introns. Locations of *Syp* locus and other surrounding genes are preserved among different species, such as rat and human (Figure 1A). Notably, *NRSE* within the first intron of *Syp* is highly conserved among closely related mammalian species (Figure 1B). Furthermore, comparison of the *Syp* upstream region also displays high conservation in proximal (approximate 0 to–600 bp) and distal (approximate−2000 to−3000 bp) regions relative to transcription start site (Figure 2). It may imply that not only *NRSE* but also the sequences of those conserved regions are involved in conferring the cell type specific expression of SYP. Therefore, for molecular engineering we have decided to use mouse BAC clone *RP23-267C15* because it encompasses the entire *Syp* locus and, thus, is likely to contain all transcriptional *cis*-elements required for recapitulation of the endogenous cell specific gene expression.

**Figure 3:**
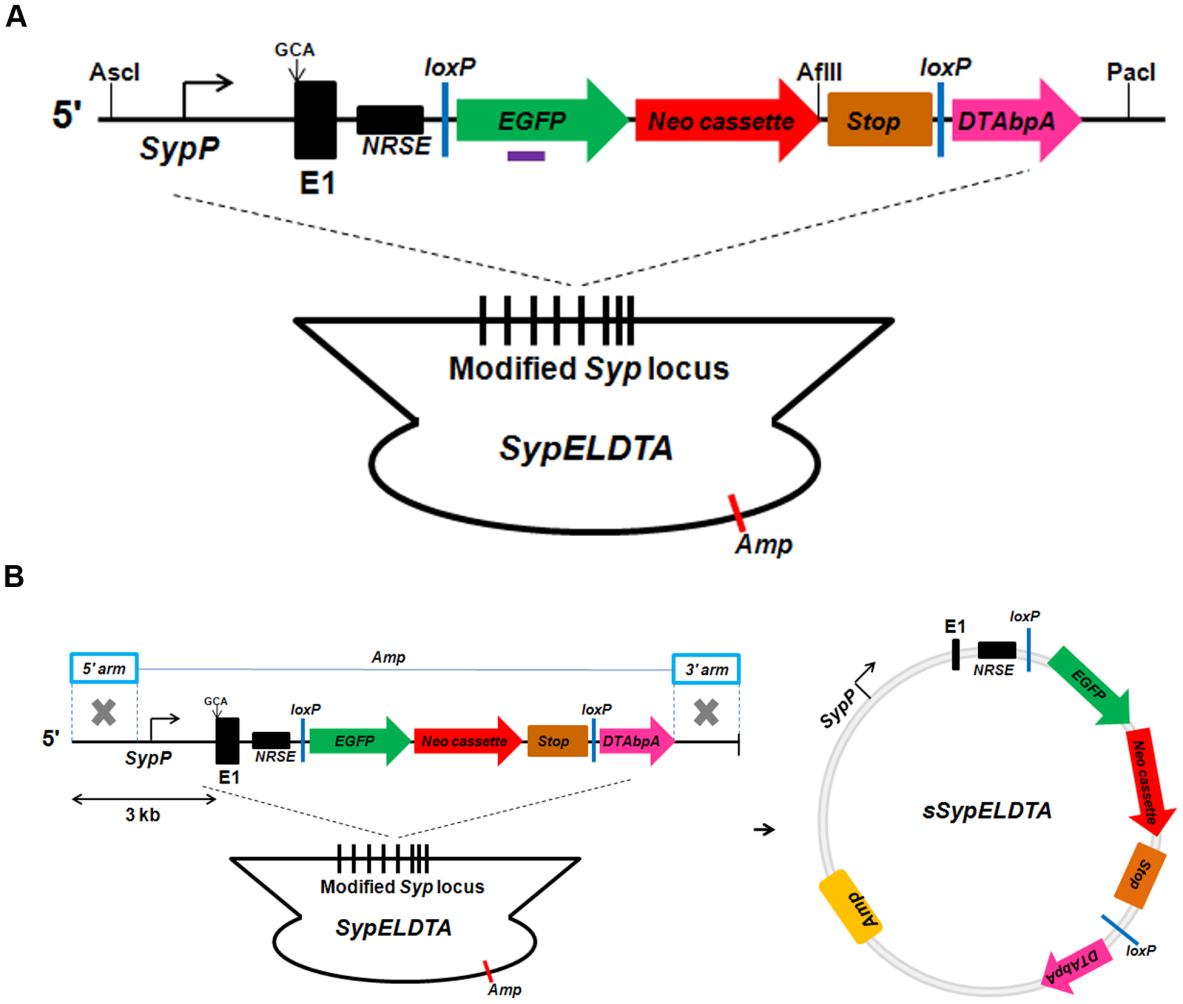
BAC transgenic constructs. (A) *SypELDTA* construct. The purple bar represents the *EGFP* probe. Restriction enzyme sites for Southern blot are indicated. (B) *sSypELDTA* construct. The fragment *SypP-loxP-EGFP-Neo cassette-Stop-loxP-DTA-bpA* from *SypELDTA* was cloned into *pGEM-T* vector. 3 kb upstream fragment of *Syp* was used to drive downstream gene expression.

First, *loxP-EGFP-Neo cassette-Stop-loxP-DTA-bpA* sequence was constructed to replace the sequence between 3' *NRSE* and exon 7 of *Syp* locus in BAC (Figure S1A). As a result, the *Syp* promoter specifically drives EGFP expression to label the SYP expressing cells. Following Cre-*loxP* mediated recombination, DTA can be expressed to ablate the SYP expressing cells. Second, the *loxP* site in the backbone of *RP23-267C15* was also replaced with *β-lactamase* sequence to avoid unintended Cre-mediated recombination due to multiple *loxP* sites (Figure S1B). The modified BAC construct is named *SypELDTA* (173 kb) (Figure 3A). It contains 121 kb and 36 kb upstream and downstream DNA sequences flanking the *Syp* gene, respectively. To evaluate applicability of shorter upstream sequence of *Syp* for cell type specific expression, the sequence *SypP*-*loxP-EGFP-Neo cassette-Stop-loxP-DTA-bpA* from *SypELDTA* was retrieved into *pGEM-T* vector. The retrieving BAC construct is named *sSypELDTA* (12 kb; Figure 3B). It contains a 3 kb upstream fragment of the *Syp* promoter region.

### BAC Transgene Function in Mammalian and Bacterial Cell Culture

Functionality of BAC construct was tested in cultured prostate cells and bacteria. *sSypELDTA* was transfected into the mouse *p53* and *Rb* deficient prostate adenocarcinoma cell lines PCN1 and PCN3, which were established from prostate carcinomas of *PB-Cre4; p53^loxP/loxP^Rb^loxP/loxP^* mice [36]. Consistent with our observation that PCN3 cells but not PCN1 cells express SYP (Figure S2A), EGFP positive cells have been observed only in PCN3 cells (Figure S2B-G) after transfection of *sSypELDTA*. Thus, 3 kb upstream fragment of *Syp* promoter region was sufficient to drive the transgene expression specifically in NE cells.

To test for Cre-*loxP* mediated recombination, *sSypELDTA* was electroporated into the *Escherichia coli* (*E. coli*) EL350 system, which contains a tightly controlled arabinose-inducible *Cre* gene. As shown by PCR, successful Cre-*loxP* mediated recombination of *sSypELDTA* in bacterial system was observed after arabinose induction (Figure S2H). The construct resulting from Cre-*loxP* mediated recombination was named *SypDTA*. To confirm that DTA expression driven by *Sy*p promoter could be detected after Cre-*loxP* mediated recombination in mouse cells, reverse transcriptase PCR (RT-PCR) was performed with different constructs transfected into PCN1 and PCN3 after Adenovirus-*Cre* (Ad-*Cre*) infection. DTA expression could be detected in *SypDTA*-infected PCN3 cells and *sSypELDTA*-infected PCN3 cells followed by Ad-*Cre* infection. Conversely, no DTA expression could be detected in PCN1 cells. Thus, DTA was expressed specifically in NE cells after Cre-*loxP* mediated recombination (Figure S2I).

### BAC Transgene Expression in *SypELDTA* Transgenic Mice

To test transgene expression *in vivo*, *sSypELDTA* and *SypELDTA* DNA were used to generate transgenic lines 141–143 and 144–148, respectively (Table 1). Based on Southern blotting and quantitative PCR (qPCR), all mice of lines 144–148 carried a single copy of transgene, while copy number of *sSypELDTA* transgene was variable among lines 141–143 (Figure S3, Table 1).

**Table 1:** Characterization of transgenic lines 141–148.

Transgene	Line	Male germ line transmission	Copy number	Transgene expression in prostate NE cells (%)*	Transgene expression in prostate non-NE cells
*sSypELDTA*	141	Yes	11	72	Yes
	142	Yes	2	81	Yes
	143	Yes	ND#	ND	Yes
*SypELDTA*	144	No	1	ND	ND
	145	Yes	1	88	No
	146	No	ND	ND	ND
	147	Yes	1	90	No
	148	Yes	1	83	No

* Percentage of EGFP; SYP double positive cells within total number of SYP positive cells was determined by double immunofluorescence staining.

# Not done.

**Figure 4:**
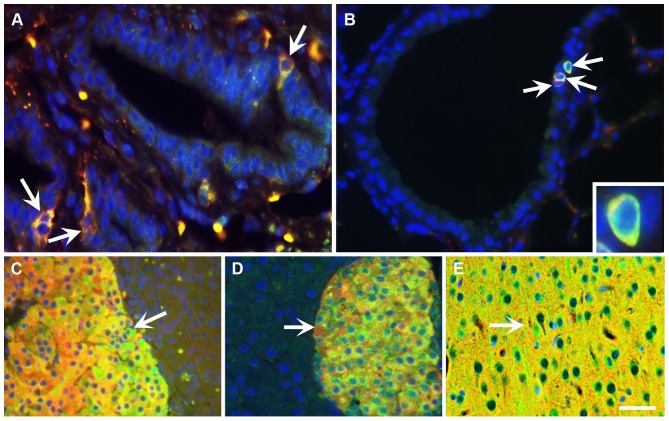
*SypELDTA* transgene expression is highly specific for SYP expressing cells. (A-E) Co-expression of EGFP (green) and SYP (red) in prostate NE cells (A), lung NE cells (B; inset: high magnification), medulla of adrenal gland (C), pancreatic islets of Langerhans (D), and brain (E) of *SypELDTA* transgenic mice. Yellow color (arrows) indicates co-localization of EGFP and SYP fluorescent signals. Counterstaining with DAPI, blue. Calibration bar: 18 µm (A), 37 µm (B), 50 µm (C-E), 8 µm (inset).

To confirm the specificity of transgene expression in transgenic mice, co-localization of transgene-derived EGFP and endogenous SYP has been determined by double immunofluorescence staining in prostate NE cells (Figure 4A). *sSypELDTA* transgenic mice had expression of transgene in non-NE epithelium cells of the prostate (Figure S4, Table 1). In contrast, no EGFP expression was detected in SYP negative cells (Figure S5, Table 2) of *SypELDTA* mice. Among 5 tested transgenic lines, the line 147 had the highest frequency (90%) of SYP positive NE cells co-expressing EGFP (Table 1). Furthermore, co-expression of EGFP and SYP in line 147 has also been observed in the lung NE cells (Figure 4B), medulla of adrenal gland (Figure 4C), islets of Langerhans in pancreas (Figure 4D), and brain (Figure 4E).

**Table 2:** Transgene expression in *SypELDTA* line 147.

Tissue	Transgene expression in SYP expressing cells (%)*	Transgene expression in non-SYP expressing cells
Brain	100	0
Lung	92	0
Medulla of adrenal gland	98	0
Islets of Langerhans	100	0

* Percentage of EGFP; SYP double positive cells within total number of SYP positive cells was determined by double immunofluorescence staining.

### DTA Expression in *EIIA-Cre; SypELDTA* Embryo

**Figure 5:**
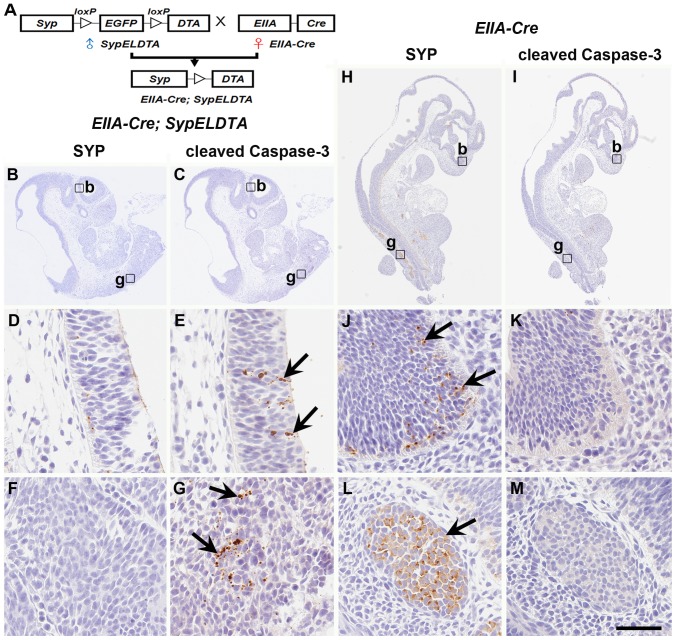
*EIIA-Cre; SypELDTA* embyros exhibit massive cell death in SYP positive cells. (A) Design of crosses between male *SypELDTA* and female *EIIA-Cre* transgenic mice. (B-M) SYP (B, D, F, H, J, L) and cleaved Caspase-3 (C, E, G, I, K, M) expression (arrows) in serial sections of *EIIA-Cre; SypELDTA* (B-G) and *EIIA-Cre* (H-M) embryos collected on gestational day 10.5. High (D-G, J-M) magnification images of brain (D, E, J, K) and dorsal root ganglion (F, G,L, M) regions shown as rectangles in low magnification images (B, C, H, I). b, brain, g, dorsal root ganglia. ABC Elite method. Hematoxylin counterstaining. Calibration bar: 950 µm (B, C, H, I), 50 µm (D-G, J-M).

To confirm that DTA expression driven by *Syp* promoter is able to ablate SYP positive cell lineage, DTA expression was examined by crossing the male *SypELDTA* mice of line 147 with female *EIIA-Cre* transgenic mice, following collection of embryos on gestational day (GD) 10.5 (Figure 5A). Adenoviral *EIIA* promoter targets expression of Cre recombinase to the early mouse embryo and Cre-mediated recombination occurs in a wide range of tissues, thereby allowing assessing effects of transgene expression in multiple organs and tissues [32]. *EIIA-Cre; SypELDTA* embryos had rare SYP positive cells in the brain and dorsal root ganglia (Figure 5B, D, F). Consistent with induction of apoptosis pathway by DTA [37], [38], a significant number of cleaved Caspase-3 positive cells were detected in the same structures (Figure 5C, E, G). The brain and dorsal root ganglia of *EIIA-Cre* littermates had abundant SYP positive cells (Figure 5H, J, L), but almost no cleaved Caspase-3 positive cells (Figure 5I, K, M). Thus, our construct was effective in conditional ablation of SYP expressing cells by DTA *in vivo*.

### Effect of Prostate Epithelium-Specific NE Cell Ablation

Modified *probasin* promoter drives expression of Cre recombinase in the prostate epithelium of postnatal mouse prostate of *PB-Cre4* transgenic mice [33], [39]. By using *PB-Cre4* mice we have previously determined that deletion of tumor suppressor genes *p53* and *Rb* results in prostate carcinomas with NE differentiation [36], [40]. We have also reported that expansion of NE cells is observed in prostate adenocarcinomas in *PB-Cre4; Pten^loxP/loxP^* mice, particularly after castration [41]. To verify that Cre recombinase under the control of *probasin* promoter is expressed in NE cells, which are concentrated in the prostate proximal region (Figure S6), Cre-mediated recombination in prostate NE cells was examined by crossing *PB-Cre4* transgenic mice [33] with R26R reporter mice [34]. The expression of β-galactosidase is possible only after Cre-mediated deletion of a stop codon flanked by *loxP* sites. Double immunofluorescence staining showed co-localized expression of β-galactosidase and SYP in the proximal region of prostatic ducts of *PB-Cre4; R26R* mice. Thus, Cre-*loxP* mediated recombination occurs in NE cell lineage after Cre expression directed by *probasin* promoter (Figure S7).

**Figure 6:**
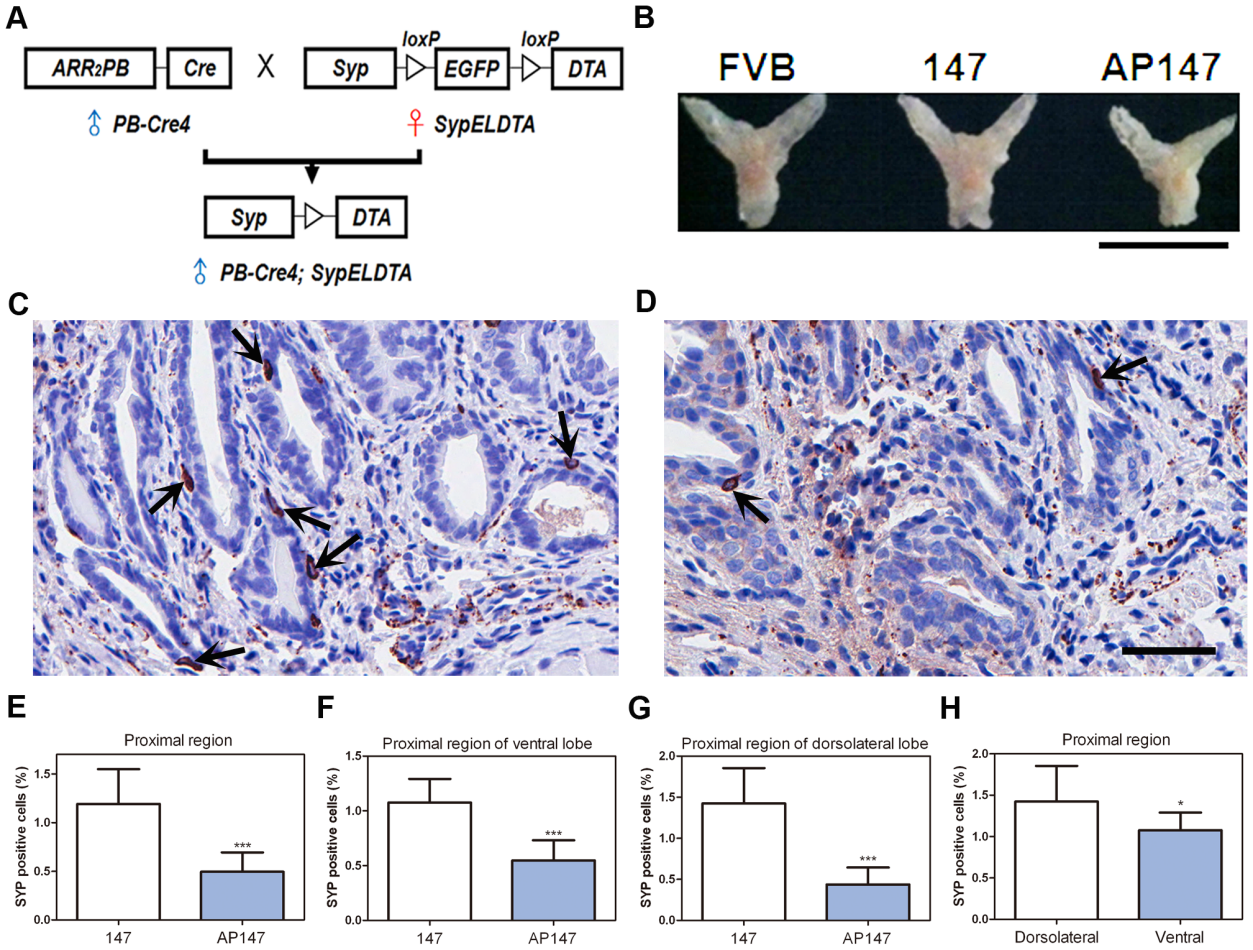
*PB-Cre4; SypELDTA* line 147 mice show decreased number of NE cells and prostate hypotrophy. (A) Design of crosses between male *PB-Cre4* and female *SypELDTA* transgenic mice resulting in male *PB-Cre4; SypELDTA* offspring with prostate epithelium-specific NE cell ablation. (B) Gross images of the prostate from wild-type FVB/N (FVB), *SypELDTA* (147), *PB-Cre4; SypELDTA* (AP147) mice. Calibration bar: 0.5 cm. (C, D) Detection of SYP positive NE cells (arrows) in the prostate proximal region of age-matched *SypELDTA* (C; n = 4) and *PB-Cre4; SypELDTA* (D; n = 4) mice. ABC Elite method. Hematoxylin counterstaining. Calibration bar: 50 µm. (E-G) Quantification of NE cells in the proximal regions of whole prostate (E), and in the proximal regions of ventral (F) and dorsolateral (G) lobes of prostates from age-matched *SypELDTA* (147; n = 4) and *PB-Cre4; SypELDTA* (AP147; n = 4) mice. (H) Quantification of NE cells in proximal regions of dorsolateral and ventral lobes of the prostates from *SypELDTA* mice (n = 4). *P<0.05. ***P<0.001. All error bars denote SD.

To evaluate whether prostate epithelium-specific NE cell ablation impacts prostate development and function, male *PB-Cre4* transgenic mice have been crossed with female *SypELDTA* (lines 147 and 148) mice to get *PB-Cre4; SypELDTA* male offspring (Figure 6A). Both lines shown similar phenotypes and line 147 has been characterized to the fullest extent. As compared to wild-type (FVB/N) and *SypELDTA* (147) age-matched controls and littermates, the size of prostate of *PB-Cre4; SypELDTA* (AP147) was smaller (Figure 6B, S8). In agreement with earlier reports of broad *PB-Cre4* transgene expression in the prostate epithelium [33], loss of floxed *EGFP* was reproducibly observed in all prostate lobes according to microdissection-PCR genotyping of AP147 (Figure S9). However, only the NE cell population was diminished by 60% in AP147 mice as compared to controls according to immunostaining for SYP in the proximal regions of prostatic ducts (147 vs. AP147: 1.19±0.34% vs. 0.5±0.19%, P<0.0001; Figure 6C-E). The decrease was observed consistently in various areas of the proximal region, such as ventral (1.07±0.22% vs. 0.55±0.18%, P<0.0001; Figure 6F) and dorsolateral (1.42±0.39% vs. 0.44±0.19%, P<0.0001; Figure 6G) lobes. In addition, the average diameter of prostatic ducts in distal regions was decreased, especially in the dorsolateral lobe (Figure S10). Interestingly, more NE cells were observed (1.42±0.39% vs. 1.07±0.22%, P = 0.0252; Figure 6H) and higher percentage of NE cells was ablated (70% vs. 49%; Figure 6F, G) in the proximal regions of prostatic ducts of dorsolateral lobes, as compared to those of ventral lobes, which could be associated with the smaller diameter of prostatic ducts in dorsolateral lobes.

Except for the size of lumens, there were no significant changes in overall morphology of prostatic ducts in the ventral and dorsolateral lobes of AP147 mice, or in either the proximal or distal regions of prostatic ducts (Figure S10). NE cell ablation did not result in any detectable changes to the extent of luminal (CK8) and basal (CK5) differentiation in the proximal and distal regions of ducts (Figure S11). No significant cell death was observed in luminal and basal cells in any of the regions (Figure S12), which implies a tight regulation of *Syp* promoter in prostate non-NE epithelium cells. Taken together, these results support the notion that NE cells play an important role in prostate development.

## Discussion

Previous report indicated that 2.6 kb but not 1.2 kb upstream fragment of *Syp* was sufficient to confer cell type specific expression [29]. Consistent with this observation, we have found high evolutionary conservation not only in the proximal (0 to –600 bp) but also distal (−2000 to −3000 bp) regions of the *Syp* gene. Based on this information, we have designed transgenic constructs preserving *NRSE* in the first intron of *Syp* gene and containing either 3 kb or 121 kb upstream regulatory sequence. While 3 kb sequence was sufficient for NE specific expression of transgene in cultured cells, it did not assure specificity of gene expression in the transgenic mice. At the same time, a longer promoter has been highly specific for NE cells. These results suggest that other *cis*-elements, farther than 3 kb upstream fragment, provide the cell type specific expression in the context of the whole organism. It is also possible that the 36 kb 3' flanking region of *Syp* might play a role in regulating cell specificity as well, similarly to other genes, such as human *tyrosine hydroxylase* [42]. Also, longer upstream and downstream regions of *Syp* locus may better insulate the *Syp* promoter from position effects by other genes at the site of transgene integration [43]. However, given NE cell specific expression of 3 kb construct after its multi-copy integration in cell culture, the latter possibility is less likely. Identification of environment driven mechanisms responsible for accurate gene expression should allow much better understanding of *Syp* regulation.

It should be noted that in addition to NE cells, SYP is also expressed in neurons of the nervous system [19], [27]. Unlike other REST regulated genes, such as *BDNF* and *GluR2* genes, *Syp* is similarly regulated in neuronal and NE cells [44]. Consistently, we have observed broad expression of *Syp* driven EGFP and DTA in mouse neurons of the brain and spinal ganglia. Thus, our model can be used for targeted ablation of NE and/or neuronal cells depending on a particular *Cre* driving promoter.

Some neuropeptides, such as calcitonin gene-related peptide (CGRP) and gastrin-releasing peptide (GRP) are expressed only in subsets of NE cells [26], [45], [46]. Therefore, theoretically it is possible that some NE cells do not express SYP. Although we were unable to find any literature supporting this possibility, our *SypELDTA* mice should allow detection of such cells by using double immunofluorescence for various NE markers before and after *Syp*-driven ablation in future.

As a proof of the utility of our model, we performed prostate epithelium-specific ablation of NE cells. The mouse prostate is composed of a series of branching ducts, each containing distal, intermediate and proximal regions relative to the urethra [47]. Each duct contains three differentiated cell types: luminal, basal and NE cells, with stem cells preferentially concentrated in the proximal region [48]–[51]. NE cells secrete a large number of neuropeptides which can be mitogenic and growth-promoting. Receptors for some of the NE products have been found to be expressed in benign prostate and/or prostate cancer. It has been proposed that the NE cells may regulate the growth, differentiation and secretory activity of the prostatic epithelium, possibly through a paracrine mechanism [11]. However, studies directly addressing role of NE cells in prostate development have been lacking.

Based on co-detection of SYP and EGFP double immunofluorescence, as well as lack of Cre-mediated recombination and cell death in prostate non-NE epithelium cells of *PB-Cre4; SypELDTA* mice, expression of *SypELDTA* has been highly specific to SYP positive prostate NE cells. Consistent with the important role of NE cells in prostate biology, prostates with decreased number of NE cells were hypotrophic, with decreased sizes of prostate lobes and reduced average diameters of prostatic ducts. Notably, the most pronounced effect of NE ablation on prostate hypotrophy was in the dorsolateral prostate, where NE cells were ablated to the greatest extent. Our study also indicates that NE cells are located in the proximal regions of the prostatic ducts, the area of preferential stem cell location. Future in-depths studies should address the kinetics of NE cell ablation effects and explore if the effect of NE cells on prostate size can be explained by their proximity to prostate stem cells.

It should be noted that in spite of 90% gene expression specificity in prostate NE cells of *SypELDTA* line 147 mice, we observed only 60% decrease in number of prostate NE cells in *PB-Cre4; SypELDTA* mice. Consistent with previous study describing compartmentalization of gene expression between prostate lobes [52], our results support a possibility that efficiency of Cre-mediated homologous recombination is different in NE cells of a particular region. We also cannot exclude that there are intrinsic differences in regulation of gene expression in individual NE cells, which may lead to the reduction of DTA expression in some of them. Thus, further improvements in design of constructs for highly efficient NE cell ablation may yield even more dramatic effect on prostate hypotrophy.

NE cell differentiation is positively correlated with prostate cancer progression, castrate-resistance and poor prognosis [12], [53]. Previous reports have indicated that NE cells can stimulate cell proliferation, invasion, and apoptosis resistance of cultured prostate cancer cells [12]. However, specific mechanisms involved in the pathogenesis of NE differentiation are not well known. Crossing *PB-Cre4; SypELDTA* transgenic mice with *Pten* knockout mice [41], [54] or other established mouse prostate cancer models with preferential NE differentiation, such as TRAMP or LADY [55], [56], should decisively determine the role of NE cells in prostate carcinogenesis. More generally, *SypELDTA* mice should be useful for studying other NE neoplasms, such as small cell lung carcinoma and NE tumors in the gastrointestinal tract [1], [2], [12].

Taken together, we have identified a region of *Syp* gene sufficient for faithful expression of genetic constructs. We have also successfully generated the *SypELDTA* mouse model suitable for flexible organ-specific detection and ablation of NE cells. This model system should provide an important tool for studies of NE cell functions in development and carcinogenesis in various tissues.

## Supporting Information

Figure S1 **Generation of the BAC targeting construct.** (A) The targeting strategy was to replace exon region of the *Syp* locus with *loxP-EGFP-Neo cassette-Stop-loxP-DTA-bpA* by homologous recombination. Exon 1 and intron 1 have been preserved because of the *NRSE* sequence, which is essential for silencing activity in non-neuronal cells. Start codon ATG in exon 1 was mutated to alanine codon GCA. *SypP*: *synaptophysin* promoter. *NRSE*: neuro-restrictive suppressor element. (B) The backbone of *RP23-267C15* (*pBACe3.6* vector) contains a *loxP* site which has been replaced with *β-lactamase* sequence by homologous recombination. The modified BAC construct was named *SypELDTA.*
(TIF)

Figure S2 **Functional testing of** ***sSypELDTA*** **in cultured prostate NE cells and** ***E. coli*** **.** (A) SYP expression in PCN1-PCN3 cells by western blotting. GAPDH, internal control. (B-G) Detection of EGFP expression by BAC transgene in prostate cancer cell line with NE differentiation (PCN3, C, E, G, arrows), but not in line without NE differentiation (PCN1, B, D, F). (B, C) Light microscopy, (D, E) green fluorescence, (F, G) merged images. Calibration bar: 200 µm (B-G). (H) Detection of Cre-*loxP* mediated recombination in *sSypELDTA* transgenic construct in the bacterial system EL350. EL350 bacteria, which contain endogenous arabinose-inducible *Cre*, were transformed with *sSypELDTA* transgenic construct and induced with arabinose. PCR genotyping was performed with F1/F2/R1 primers without (lane 2) and with (lane 3) arabinose induction. 256 bp and 617 bp fragments are diagnostic for internal control (primer F2/R1) and Cre-mediated recombination (primer F1/R1), respectively. Lane 1: marker (M). (I) Detection of DTA expression by *sSypELDTA* transgenic construct in the prostate cancer cell lines by RT-PCR. Lane 1: marker (M); Lane 2 (without RT) and Lane 3 (with RT): no transfection; Lane 4 (without RT) and Lane 5 (with RT): *PGKDTAbpA* transfection; Lane 6 (without RT) and Lane 7 (with RT): *sSypELDTA* transfection; Lane 8 (without RT) and Lane 9 (with RT): *sSypELDTA* transfection followed by Ad-*Cre* infection; Lane 10 (without RT) and Lane 11 (with RT): *SypDTA* transfection. 657 bp fragment is diagnostic for DTA mRNA; RT, reverse transcriptase.
(TIF)

Figure S3 **The transgene copy number in mice of** ***sSypELDTA*** **lines 141-143 and** ***SypELDTA*** **lines 144-148.** (A) Experimental design. (B) Southern blot analysis of transgenic line 141. Genomic DNA digested with restriction enzymes AscI (Lane 2), PacI (Lane 3), and AscI, PacI and AflII (Lane 4). Lane 1: DNA marker (M). AflII but not AscI and PacI restriction sites are present within the transgene. 8,466 bp band in lane 4 is diagnostic for multiple transgene copies integrated into a single genomic site, whereas top band (arrow) is diagnostic for single copy of transgene. *EGFP*, DNA probe. (C) Quantification of transgene copy number in transgenic lines by quantitative PCR. Transgenic line 141 carrying 11 copies of transgene was used as a reference to estimate copy number of other lines. (D) Genotyping analysis of transgenic line 147. The upper bands are diagnostic for *EGFP* (lane 2, E, 346 bp), *Neomycin* (lane 3, N, 342 bp), and *DTA* (lane 4, D, 348 bp) fragments of transgene. 196 bp band in lanes 2-4 is internal control (endogenous *Rb*). Lane 1: DNA marker (M).
(TIF)

Figure S4 **Transgene expression in** ***sSypELDTA*** **lines 141-143.** (A-H) Detection of EGFP (B, D, F, H, green) and SYP (C, D, G, H, red) expression (arrows) in non-NE (A-D) and NE cells (E-H) of the prostate epithelium. Yellow color in overlay (D, H) indicates co-localization of EGFP and SYP fluorescent signals. Counterstaining with DAPI (A, D, E, H, blue). Calibration bar: 50 µm (A-D), 18 µm (E-H).
(TIF)

Figure S5 ***SypELDTA*** **transgene expression has high specificity in SYP expressing cells.** (A-T) Detection of EGFP (B, D, F, H, J, L, N, P, R, T, green) and SYP (C, D, G, H, K, L, O, P, S, T, red) expression (arrows) in prostate NE cells (A-D), lung NE cells (E-H), medulla of adrenal gland (I-L), islets of Langerhans in pancreas (M-P), and brain (Q-T) of *SypELDTA* line 147 transgenic mice. Yellow color in overlay (D, H, L, P, T) indicates co-localization of EGFP and SYP fluorescent signals. Counterstaining with DAPI (A, D, E, H, I, L, M, P, Q, T, blue). Calibration bar: 25 µm (A-D), 50 µm (E-T).
(TIF)

Figure S6 **NE cells are mostly located in the proximal region of prostatic ducts.** (A, B) SYP expression in NE cells in proximal (A) and distal (B) regions of prostatic ducts of the prostate (n = 6). NE cells and nerve terminals are indicated by arrows and arrowheads, respectively. Calibration bar: 50 µm (A), 100 µm (B). (C) Quantification of SYP positive NE cells. Distal regions of prostatic ducts contain no NE cells. Error bar denotes SD.
(TIF)

Figure S7 **Cre recombinase under the control of** ***probasin*** **promoter is expressed in prostate NE cells.** (A-D) Detection of SYP (B, D, green) and β-galactosidase (C, D, red, indicative of Cre-*loxP* mediated recombination) expression (arrows) in the prostate NE cells in *PB-Cre4; R26R* mice harboring *Probasin*-*Cre* and *lacZ* reporter gene. Yellow color in overlay (D) indicates co-localization of SYP and β-galactosidase fluorescent signals. Counterstaining with DAPI (A, D, blue). Calibration bar: 50 µm (A-D).
(TIF)

Figure S8 **Reduced size of prostate lobes in** ***PB-Cre4; SypELDTA*** **mice.** (A-D) Quantification of size of anterior (A), dorsal(B), lateral (C), and ventral (D) lobes among age-matched FVB/N (FVB, n = 4), *SypELDTA* (147; n = 4), and *PB-Cre4; SypELDTA* (AP147; n = 4) mice. *P<0.05. **P<0.01. Error bar denotes SD.
(TIF)

Figure S9 ***PB-Cre*** **drives Cre-** ***loxP*** **recombination in the prostate of** ***PB-Cre4; SypELDTA*** **mice.** (A-F) Microdissection-PCR. Proximal (A) and distal (ventral, B, dorsolateral, C, and anterior, D) regions of prostatic ducts and the muscular layer of the prostate (E) of age-matched *SypELDTA* (147) and *PB-Cre4; SypELDTA* (AP147) mice before and after microdissection. Hematoxylin and eosin. Calibration bar: 50 µm (A-E). (F) PCR design and detection of Cre-*loxP* mediated recombination in microdissected proximal region (P, lane 2 and 7), and ventral (V, lane 3 and 8), dorsolateral (DL, lane 4 and 9), anterior (A, lane 5 and 10) distal regions, and muscular layer (ML, lane 6) of prostates from *SypELDTA*(lanes 2-5) and *PB-Cre4; SypELDTA* (lanes 6-10) mice. 346 bp fragment is generated with primers F1and R1and is diagnostic for *EGFP* (present before Cre-loxP mediated recombination). 196 bp fragment (endogenous *Rb*) is internal control. Lane 1: marker (M).
(TIF)

Figure S10 **NE cell ablation results in proportional reduction of prostatic duct diameters in distal regions.** (A-I) Histology (A, B, D, E, G, H) and quantification of diameters (C, F, I) of proximal (A, B, C) and distal (ventral, D, E, F, and dorsolateral, G, H, I) regions of prostatic ducts of age-matched *SypELDTA* (147; n = 6; A, D, G) and *PB-Cre4; SypELDTA* (AP147, n = 6; B, E, H) mice. Hematoxylin and eosin. Calibration bar: 50 µm (A, B), 100 µm (D, E, G, H). *P<0.05. **P<0.01. All error bars denote SD.
(TIF)

Figure S11 **NE cell ablation does not affect luminal (CK8+) or basal (CK5+) cell differentiation.** (A-L) Detection of CK8 (A-F) and CK5 (G-L) expression (brown) in epithelial cells of proximal (A, D, G, J) and distal (ventral, B, E, H, K, and dorsolateral, C, F, I, L) regions of prostatic ducts in age-matched *SypELDTA* (147, A-C, G-I) and *PB-Cre4; SypELDTA* (AP147, D-F, J-L) mice. ABC Elite method. Hematoxylin counterstaining. Calibration bar: 50 µm (A-L).
(TIF)

Figure S12 **No significant cell death is observed in prostate epithelium non-NE cells in** ***PB-Cre4; SypELDTA*** **mice.** (A-F) cleaved Caspase-3 expression in proximal (A, B), and distal (ventral, C, D, and dorsolateral, E, F) regions of prostatic ducts in age-matched *SypELDTA* (147, A, C, E) and *PB-Cre4; SypELDTA* (AP147, B, D, F) mice. The staining of embryonic dorsal root ganglia (Figure 5) served as a positive control for cleaved Caspase-3 immunostaining. ABC Elite method. Hematoxylin counterstaining. Calibration bar: 50 µm (A-F).
(TIF)

Materials and Methods S1 **Detailed protocol descriptions for generation of the targeting construct and BAC recombineering, genotyping, cell culture experiments, transgene copy number quantification, antibody sources and dilutions of immunohistochemical analyses, and microdissection-polymerase chain reaction.**
(DOCX)

## References

[1] Montuenga LM, Guembe L, Burrell MA, Bodegas ME, Calvo A, et al (2003) The diffuse endocrine system: from embryogenesis to carcinogenesis. Prog Histochem Cytochem 38: 155–272. 12756892 10.1016/s0079-6336(03)80004-9

[2] Linnoila RI (2006) Functional facets of the pulmonary neuroendocrine system. Lab Invest 86: 425–444. 16568108 10.1038/labinvest.3700412

[3] Ahren B (2000) Autonomic regulation of islet hormone secretion--implications for health and disease. Diabetologia 43: 393–410. 10819232 10.1007/s001250051322

[4] Koh DS, Cho JH, Chen L (2012) Paracrine Interactions Within Islets of Langerhans. J Mol Neurosci 48: 429–440. 22528452 10.1007/s12031-012-9752-2

[5] Dockray G (2003) Making sense of gut contents. Scand J Gastroenterol 38: 451–455. 12795452 10.1080/00365520310000799

[6] Kuliczkowska-Plaksej J, Milewicz A, Jakubowska J (2012) Neuroendocrine control of metabolism. Gynecol Endocrinol 28 Suppl 127–32. 22394301 10.3109/09513590.2012.651930

[7] Tachibana T (1995) The Merkel cell: recent findings and unresolved problems. Arch Histol Cytol 58: 379–396. 8562130 10.1679/aohc.58.379

[8] Mravec B (2005) A new focus on interoceptive properties of adrenal medulla. Auton Neurosci 120: 10–17. 15927540 10.1016/j.autneu.2005.04.005

[9] de Diego AM, Gandia L, Garcia AG (2008) A physiological view of the central and peripheral mechanisms that regulate the release of catecholamines at the adrenal medulla. Acta Physiol (Oxf) 192: 287–301. 18005392 10.1111/j.1748-1716.2007.01807.x

[10] Douglas SA, Sreenivasan D, Carman FH, Bunn SJ (2010) Cytokine interactions with adrenal medullary chromaffin cells. Cell Mol Neurobiol 30: 1467–1475. 21088883 10.1007/s10571-010-9593-x PMC11498763

[11] Abrahamsson PA (1999) Neuroendocrine differentiation in prostatic carcinoma. Prostate 39: 135–148. 10221570 10.1002/(sici)1097-0045(19990501)39:2<135::aid-pros9>3.0.co;2-s

[12] Sun Y, Niu J, Huang J (2009) Neuroendocrine differentiation in prostate cancer. Am J Transl Res 1: 148–162. 19956427 PMC2776313

[13] Gustafsson BI, Kidd M, Modlin IM (2008) Neuroendocrine tumors of the diffuse neuroendocrine system. Curr Opin Oncol 20: 1–12. 18043250 10.1097/CCO.0b013e3282f1c595

[14] Cheng CY, Nikitin AY (2011) Neuroendocrine cells: potential cells of origin for small cell lung carcinoma. Cell Cycle 10: 3629–3630. 10.4161/cc.10.21.18034 PMC3356821 22024916

[15] Gazdar AF, Helman LJ, Israel MA, Russell EK, Linnoila RI, et al (1988) Expression of neuroendocrine cell markers L-dopa decarboxylase, chromogranin A, and dense core granules in human tumors of endocrine and nonendocrine origin. Cancer Res 48: 4078–4082. 3383200

[16] Schmechel D, Marangos PJ, Brightman M (1978) Neurone-specific enolase is a molecular marker for peripheral and central neuroendocrine cells. Nature 276: 834–836. 31568 10.1038/276834a0

[17] Jin L, Hemperly JJ, Lloyd RV (1991) Expression of neural cell adhesion molecule in normal and neoplastic human neuroendocrine tissues. Am J Pathol 138: 961–969. 2012179 PMC1886094

[18] Cadieux A, Springall DR, Mulderry PK, Rodrigo J, Ghatei MA, et al (1986) Occurrence, distribution and ontogeny of CGRP immunoreactivity in the rat lower respiratory tract: effect of capsaicin treatment and surgical denervations. Neuroscience 19: 605–627. 3490633 10.1016/0306-4522(86)90285-x

[19] Wiedenmann B, Franke WW, Kuhn C, Moll R, Gould VE (1986) Synaptophysin: a marker protein for neuroendocrine cells and neoplasms. Proc Natl Acad Sci U S A 83: 3500–3504. 3010302 10.1073/pnas.83.10.3500 PMC323544

[20] Haimoto H, Takahashi Y, Koshikawa T, Nagura H, Kato K (1985) Immunohistochemical localization of gamma-enolase in normal human tissues other than nervous and neuroendocrine tissues. Lab Invest 52: 257–263. 3974199

[21] Schmechel DE (1985) Gamma-subunit of the glycolytic enzyme enolase: nonspecific or neuron specific? Lab Invest 52: 239–242. 3974198

[22] Seshi B, True L, Carter D, Rosai J (1988) Immunohistochemical characterization of a set of monoclonal antibodies to human neuron-specific enolase. Am J Pathol 131: 258–269. 3282444 PMC1880593

[23] Kaufmann O, Georgi T, Dietel M (1997) Utility of 123C3 monoclonal antibody against CD56 (NCAM) for the diagnosis of small cell carcinomas on paraffin sections. Hum Pathol 28: 1373–1378. 9416693 10.1016/s0046-8177(97)90226-4

[24] Lantuejoul S, Moro D, Michalides RJ, Brambilla C, Brambilla E (1998) Neural cell adhesion molecules (NCAM) and NCAM-PSA expression in neuroendocrine lung tumors. Am J Surg Pathol 22: 1267–1276. 9777989 10.1097/00000478-199810000-00012

[25] Jensen SM, Gazdar AF, Cuttitta F, Russell EK, Linnoila RI (1990) A comparison of synaptophysin, chromogranin, and L-dopa decarboxylase as markers for neuroendocrine differentiation in lung cancer cell lines. Cancer Res 50: 6068–6074. 2168288

[26] Weichselbaum M, Sparrow MP, Hamilton EJ, Thompson PJ, Knight DA (2005) A confocal microscopic study of solitary pulmonary neuroendocrine cells in human airway epithelium. Respir Res 6: 115. 16216130 10.1186/1465-9921-6-115 PMC1277851

[27] Gould VE, Wiedenmann B, Lee I, Schwechheimer K, Dockhorn-Dworniczak B, et al (1987) Synaptophysin expression in neuroendocrine neoplasms as determined by immunocytochemistry. Am J Pathol 126: 243–257. 3103452 PMC1899573

[28] Sudhof TC, Lottspeich F, Greengard P, Mehl E, Jahn R (1987) A synaptic vesicle protein with a novel cytoplasmic domain and four transmembrane regions. Science 238: 1142–1144. 3120313 10.1126/science.3120313

[29] Bargou RC, Leube RE (1991) The synaptophysin-encoding gene in rat and man is specifically transcribed in neuroendocrine cells. Gene 99: 197–204. 1902431 10.1016/0378-1119(91)90127-w

[30] Lietz M, Hohl M, Thiel G (2003) RE-1 silencing transcription factor (REST) regulates human synaptophysin gene transcription through an intronic sequence-specific DNA-binding site. Eur J Biochem 270: 2–9. 12492469 10.1046/j.1432-1033.2003.03360.x

[31] Ivanova A, Signore M, Caro N, Greene ND, Copp AJ, et al (2005) In vivo genetic ablation by Cre-mediated expression of diphtheria toxin fragment A. Genesis. 43: 129–135. 10.1002/gene.20162 PMC2233880 16267821

[32] Lakso M, Pichel JG, Gorman JR, Sauer B, Okamoto Y, et al (1996) Efficient in vivo manipulation of mouse genomic sequences at the zygote stage. Proc Natl Acad Sci U S A 93: 5860–5865. 8650183 10.1073/pnas.93.12.5860 PMC39152

[33] Wu X, Wu J, Huang J, Powell WC, Zhang J, et al (2001) Generation of a prostate epithelial cell-specific Cre transgenic mouse model for tissue-specific gene ablation. Mech Dev 101: 61–69. 11231059 10.1016/s0925-4773(00)00551-7

[34] Soriano P (1999) Generalized lacZ expression with the ROSA26 Cre reporter strain. Nat Genet 21: 70–71. 9916792 10.1038/5007

[35] Nikitin A, Lee WH (1996) Early loss of the retinoblastoma gene is associated with impaired growth inhibitory innervation during melanotroph carcinogenesis in Rb+/− mice. Genes Dev 10: 1870–1879. 8756345 10.1101/gad.10.15.1870

[36] Zhou Z, Flesken-Nikitin A, Corney DC, Wang W, Goodrich DW, et al (2006) Synergy of p53 and Rb deficiency in a conditional mouse model for metastatic prostate cancer. Cancer Res 66: 7889–7898. 16912162 10.1158/0008-5472.CAN-06-0486

[37] Chang MP, Baldwin RL, Bruce C, Wisnieski BJ (1989) Second cytotoxic pathway of diphtheria toxin suggested by nuclease activity. Science 246: 1165–1168. 2531465 10.1126/science.2531465

[38] Chang MP, Bramhall J, Graves S, Bonavida B, Wisnieski BJ (1989) Internucleosomal DNA cleavage precedes diphtheria toxin-induced cytolysis. Evidence that cell lysis is not a simple consequence of translation inhibition. J Biol Chem 264: 15261–15267. 2768263

[39] Chen Z, Trotman LC, Shaffer D, Lin HK, Dotan ZA, et al (2005) Crucial role of p53-dependent cellular senescence in suppression of Pten-deficient tumorigenesis. Nature 436: 725–730. 16079851 10.1038/nature03918 PMC1939938

[40] Zhou Z, Flesken-Nikitin A, Nikitin AY (2007) Prostate cancer associated with p53 and Rb deficiency arises from the stem/progenitor cell-enriched proximal region of prostatic ducts. Cancer Res 67: 5683–5690. 17553900 10.1158/0008-5472.CAN-07-0768

[41] Liao CP, Zhong C, Saribekyan G, Bading J, Park R, et al (2007) Mouse models of prostate adenocarcinoma with the capacity to monitor spontaneous carcinogenesis by bioluminescence or fluorescence. Cancer Res 67: 7525–7533. 17671224 10.1158/0008-5472.CAN-07-0668

[42] Wong SC, Moffat MA, Coker GT, Merlie JP, O'Malley KL (1995) The 3' flanking region of the human tyrosine hydroxylase gene directs reporter gene expression in peripheral neuroendocrine tissues. J Neurochem 65: 23–31. 7790865 10.1046/j.1471-4159.1995.65010023.x

[43] Yang XW, Gong S (2005) An overview on the generation of BAC transgenic mice for neuroscience research. Curr Protoc Neurosci Chapter 5: Unit 5 20. 10.1002/0471142301.ns0520s31 18428622

[44] Hohl M, Thiel G (2005) Cell type-specific regulation of RE-1 silencing transcription factor (REST) target genes. Eur J Neurosci 22: 2216–2230. 16262660 10.1111/j.1460-9568.2005.04404.x

[45] di Sant'Agnese PA, de Mesy Jensen KL, Ackroyd RK (1989) Calcitonin, katacalcin, and calcitonin gene-related peptide in the human prostate. An immunocytochemical and immunoelectron microscopic study. Arch Pathol Lab Med 113: 790–796. 2787149

[46] Sunday ME, Kaplan LM, Motoyama E, Chin WW, Spindel ER (1988) Gastrin-releasing peptide (mammalian bombesin) gene expression in health and disease. Lab Invest 59: 5–24. 2839735

[47] Sugimura Y, Cunha GR, Donjacour AA (1986) Morphogenesis of ductal networks in the mouse prostate. Biol Reprod 34: 961–971. 3730488 10.1095/biolreprod34.5.961

[48] Salm SN, Burger PE, Coetzee S, Goto K, Moscatelli D, et al (2005) TGF-{beta} maintains dormancy of prostatic stem cells in the proximal region of ducts. J Cell Biol 170: 81–90. 15983059 10.1083/jcb.200412015 PMC2171389

[49] Tsujimura A, Koikawa Y, Salm S, Takao T, Coetzee S, et al (2002) Proximal location of mouse prostate epithelial stem cells: a model of prostatic homeostasis. J Cell Biol 157: 1257–1265. 12082083 10.1083/jcb.200202067 PMC2173539

[50] Wang GM, Kovalenko B, Wilson EL, Moscatelli D (2007) Vascular density is highest in the proximal region of the mouse prostate. Prostate 67: 968–975. 17440972 10.1002/pros.20582 PMC2430188

[51] Leong KG, Wang BE, Johnson L, Gao WQ (2008) Generation of a prostate from a single adult stem cell. Nature 456: 804–808. 18946470 10.1038/nature07427

[52] Abbott DE, Pritchard C, Clegg NJ, Ferguson C, Dumpit R, et al (2003) Expressed sequence tag profiling identifies developmental and anatomic partitioning of gene expression in the mouse prostate. Genome Biol 4: R79. 14659016 10.1186/gb-2003-4-12-r79 PMC329418

[53] Debes JD, Tindall DJ (2004) Mechanisms of androgen-refractory prostate cancer. N Engl J Med 351: 1488–1490. 15470210 10.1056/NEJMp048178

[54] Wang S, Gao J, Lei Q, Rozengurt N, Pritchard C, et al (2003) Prostate-specific deletion of the murine Pten tumor suppressor gene leads to metastatic prostate cancer. Cancer Cell 4: 209–221. 14522255 10.1016/s1535-6108(03)00215-0

[55] Kaplan-Lefko PJ, Chen TM, Ittmann MM, Barrios RJ, Ayala GE, et al (2003) Pathobiology of autochthonous prostate cancer in a pre-clinical transgenic mouse model. Prostate 55: 219–237. 12692788 10.1002/pros.10215

[56] Masumori N, Thomas TZ, Chaurand P, Case T, Paul M, et al (2001) A probasin-large T antigen transgenic mouse line develops prostate adenocarcinoma and neuroendocrine carcinoma with metastatic potential. Cancer Res 61: 2239–2249. 11280793

