# Supplementary figures and images for: Detection and Organ-Specific Ablation of Neuroendocrine Cells by Synaptophysin Locus-Based BAC Cassette in Transgenic Mice
Source: PLoS One. 2013 Apr 22;8(4):e60905. doi: 10.1371/journal.pone.0060905 (PMC3632533; doi:10.1371/journal.pone.0060905)

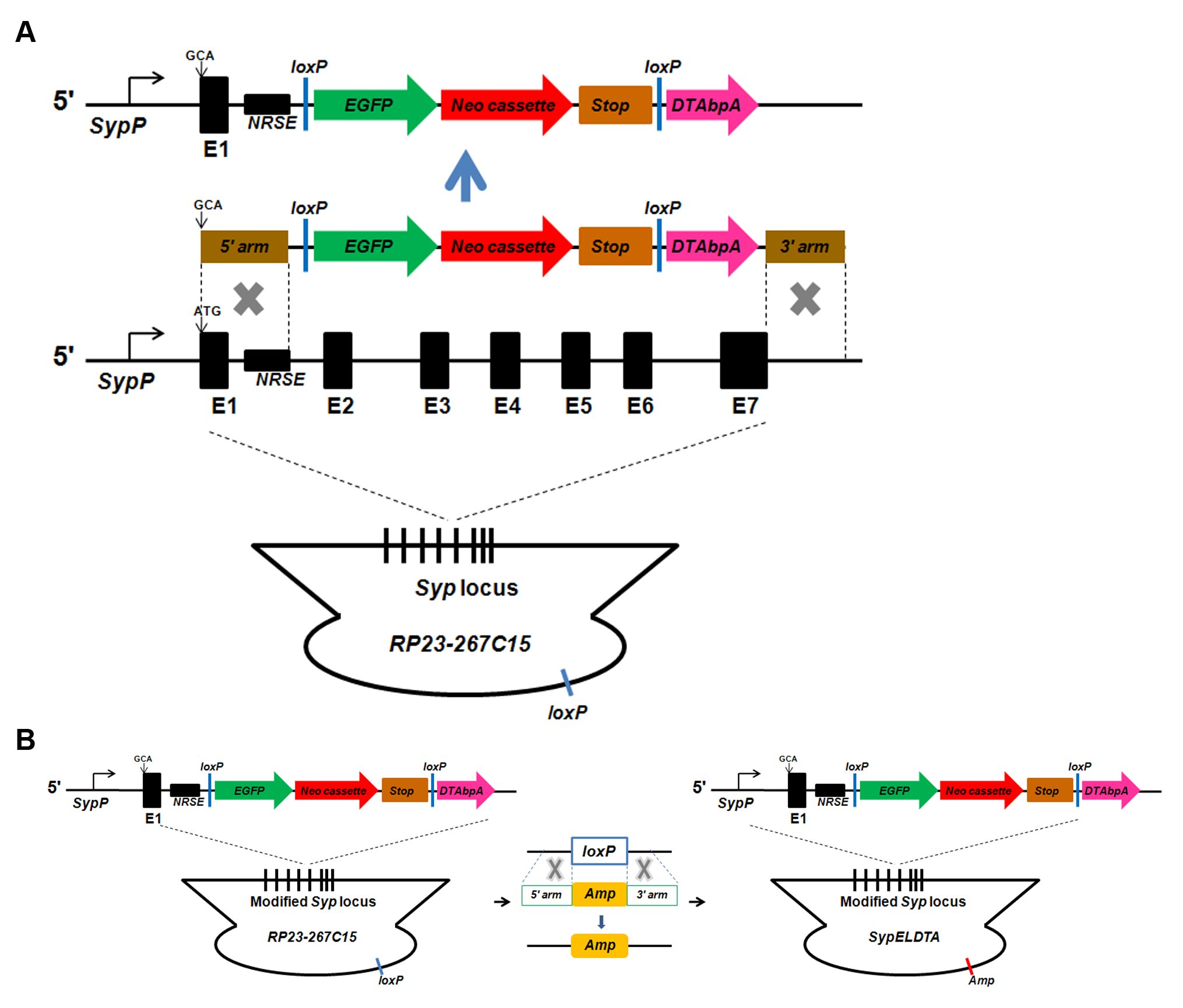

Supplement: Figure S1 — Generation of the BAC targeting construct. (A) The targeting strategy was to replace exon region of the Syp locus with loxP-EGFP-Neo cassette-Stop-loxP-DTA-bpA by homologous recombination. Exon 1 and intron 1 have been preserved because of the NRSE sequence, which is essential for silencing activity in non-neuronal cells. Start codon ATG in exon 1 was mutated to alanine codon GCA. SypP: synaptophysin promoter. NRSE: neuro-restrictive suppressor element. (B) The backbone of RP23-267C15 (pBACe3.6 vector) contains a loxP site which has been replaced with β-lactamase sequence by homologous recombination. The modified BAC construct was named SypELDTA. (TIF) [file pone.0060905.s001.tif]

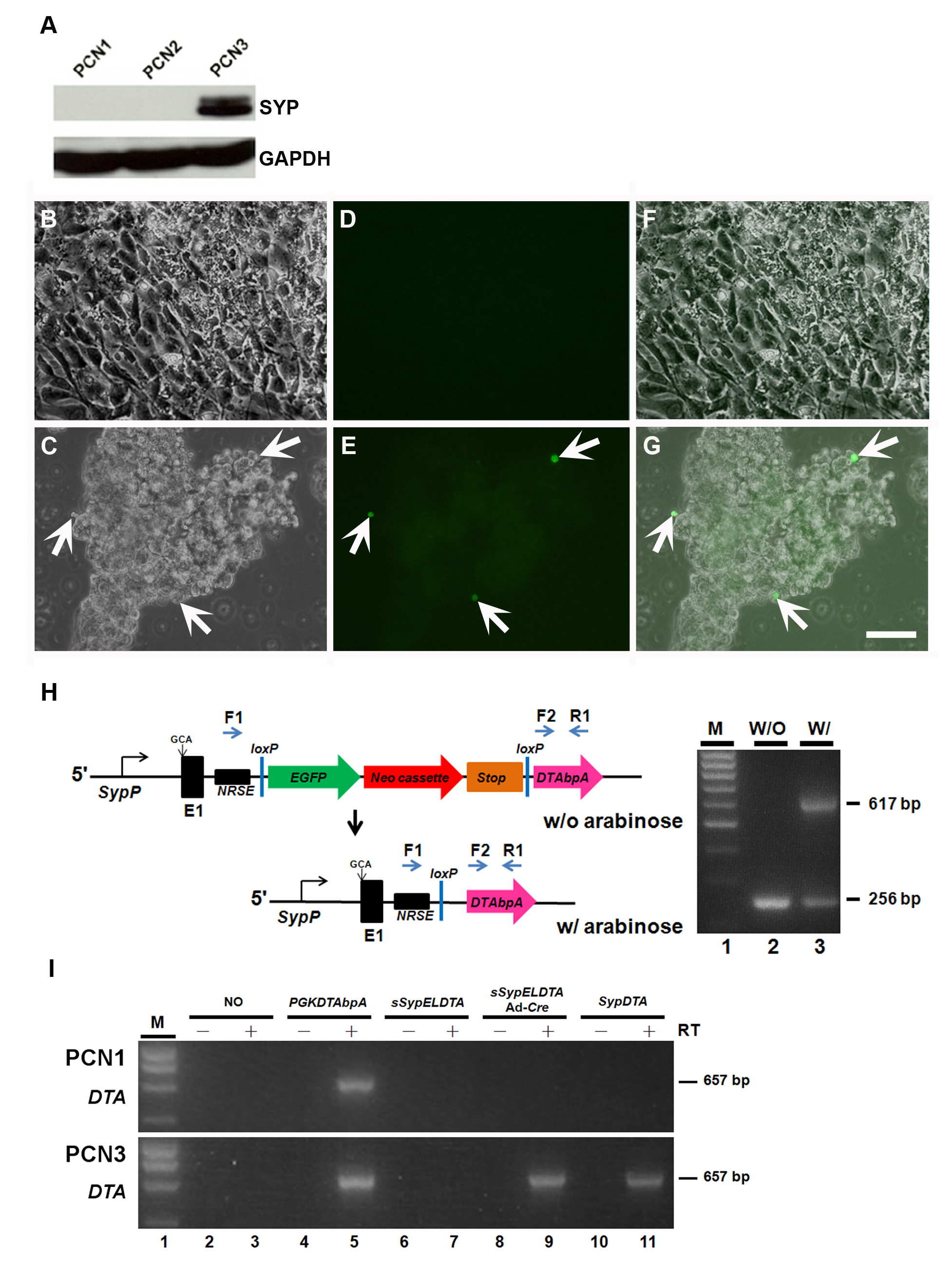

Supplement: Figure S2 — Functional testing of sSypELDTA in cultured prostate NE cells and E. coli . (A) SYP expression in PCN1-PCN3 cells by western blotting. GAPDH, internal control. (B-G) Detection of EGFP expression by BAC transgene in prostate cancer cell line with NE differentiation (PCN3, C, E, G, arrows), but not in line without NE differentiation (PCN1, B, D, F). (B, C) Light microscopy, (D, E) green fluorescence, (F, G) merged images. Calibration bar: 200 µm (B-G). (H) Detection of Cre-loxP mediated recombination in sSypELDTA transgenic construct in the bacterial system EL350. EL350 bacteria, which contain endogenous arabinose-inducible Cre, were transformed with sSypELDTA transgenic construct and induced with arabinose. PCR genotyping was performed with F1/F2/R1 primers without (lane 2) and with (lane 3) arabinose induction. 256 bp and 617 bp fragments are diagnostic for internal control (primer F2/R1) and Cre-mediated recombination (primer F1/R1), respectively. Lane 1: marker (M). (I) Detection of DTA expression by sSypELDTA transgenic construct in the prostate cancer cell lines by RT-PCR. Lane 1: marker (M); Lane 2 (without RT) and Lane 3 (with RT): no transfection; Lane 4 (without RT) and Lane 5 (with RT): PGKDTAbpA transfection; Lane 6 (without RT) and Lane 7 (with RT): sSypELDTA transfection; Lane 8 (without RT) and Lane 9 (with RT): sSypELDTA transfection followed by Ad-Cre infection; Lane 10 (without RT) and Lane 11 (with RT): SypDTA transfection. 657 bp fragment is diagnostic for DTA mRNA; RT, reverse transcriptase. (TIF) [file pone.0060905.s002.tif]

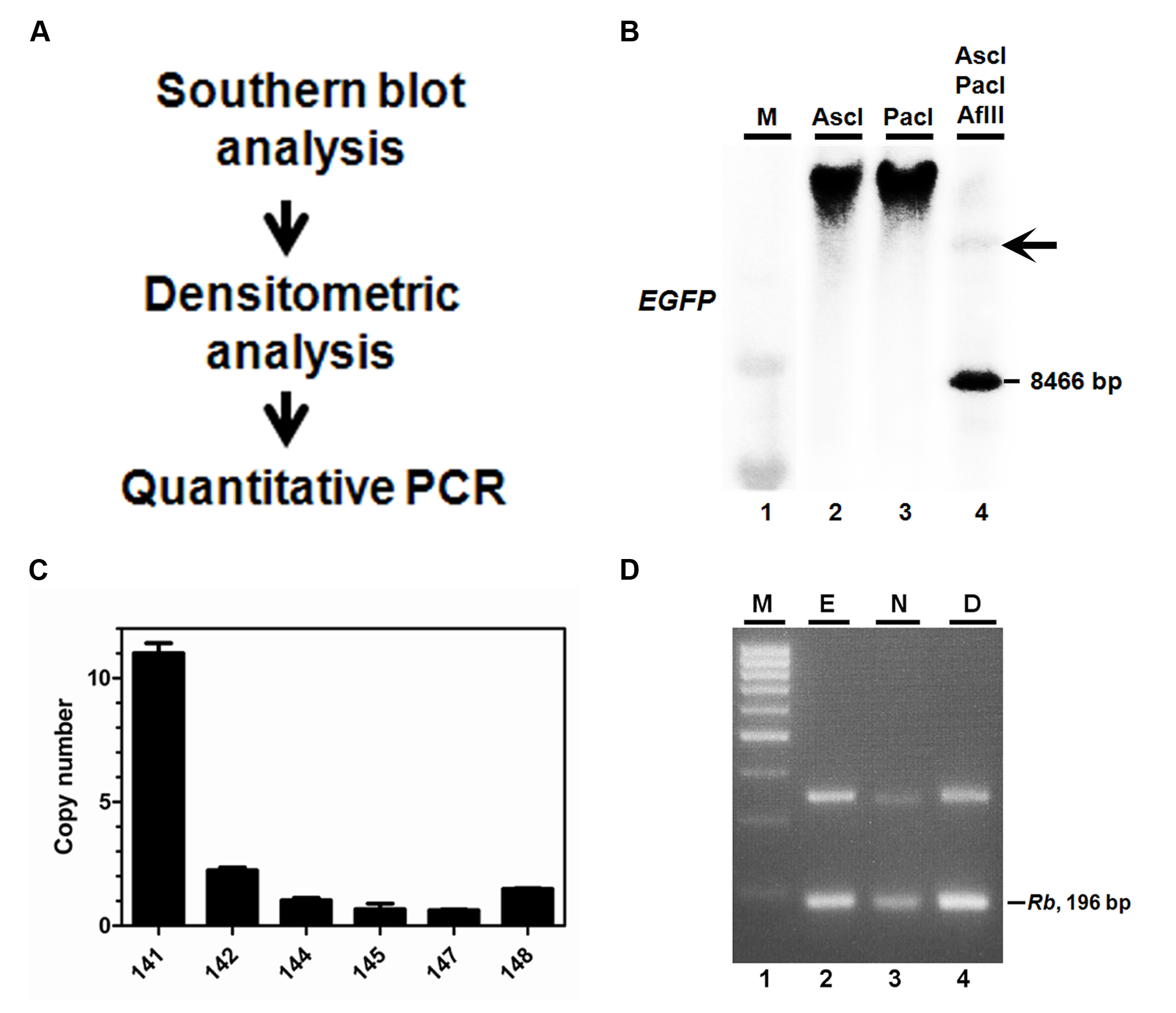

Supplement: Figure S3 — The transgene copy number in mice of sSypELDTA lines 141-143 and SypELDTA lines 144-148. (A) Experimental design. (B) Southern blot analysis of transgenic line 141. Genomic DNA digested with restriction enzymes AscI (Lane 2), PacI (Lane 3), and AscI, PacI and AflII (Lane 4). Lane 1: DNA marker (M). AflII but not AscI and PacI restriction sites are present within the transgene. 8,466 bp band in lane 4 is diagnostic for multiple transgene copies integrated into a single genomic site, whereas top band (arrow) is diagnostic for single copy of transgene. EGFP, DNA probe. (C) Quantification of transgene copy number in transgenic lines by quantitative PCR. Transgenic line 141 carrying 11 copies of transgene was used as a reference to estimate copy number of other lines. (D) Genotyping analysis of transgenic line 147. The upper bands are diagnostic for EGFP (lane 2, E, 346 bp), Neomycin (lane 3, N, 342 bp), and DTA (lane 4, D, 348 bp) fragments of transgene. 196 bp band in lanes 2-4 is internal control (endogenous Rb). Lane 1: DNA marker (M). (TIF) [file pone.0060905.s003.tif]

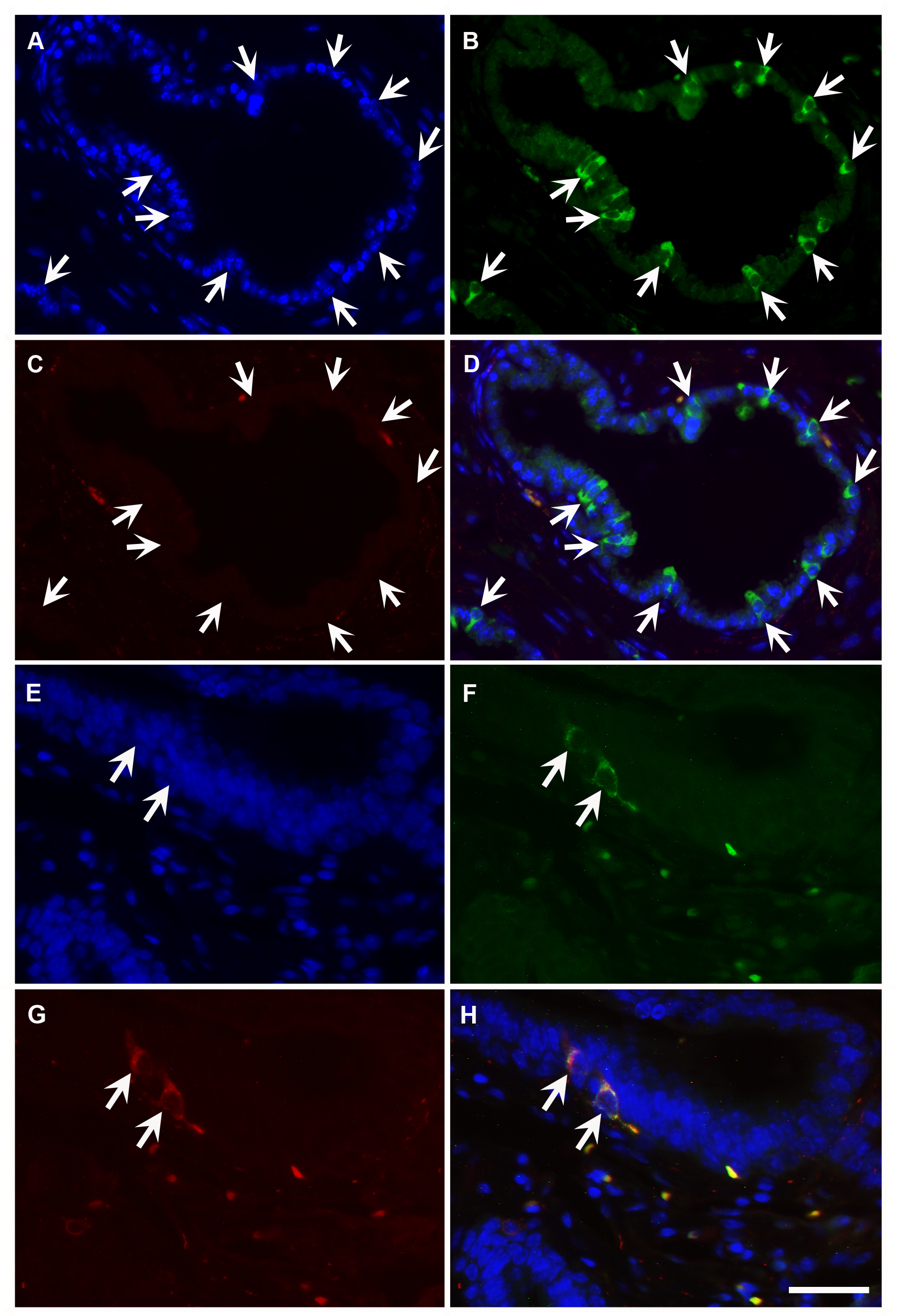

Supplement: Figure S4 — Transgene expression in sSypELDTA lines 141-143. (A-H) Detection of EGFP (B, D, F, H, green) and SYP (C, D, G, H, red) expression (arrows) in non-NE (A-D) and NE cells (E-H) of the prostate epithelium. Yellow color in overlay (D, H) indicates co-localization of EGFP and SYP fluorescent signals. Counterstaining with DAPI (A, D, E, H, blue). Calibration bar: 50 µm (A-D), 18 µm (E-H). (TIF) [file pone.0060905.s004.tif]

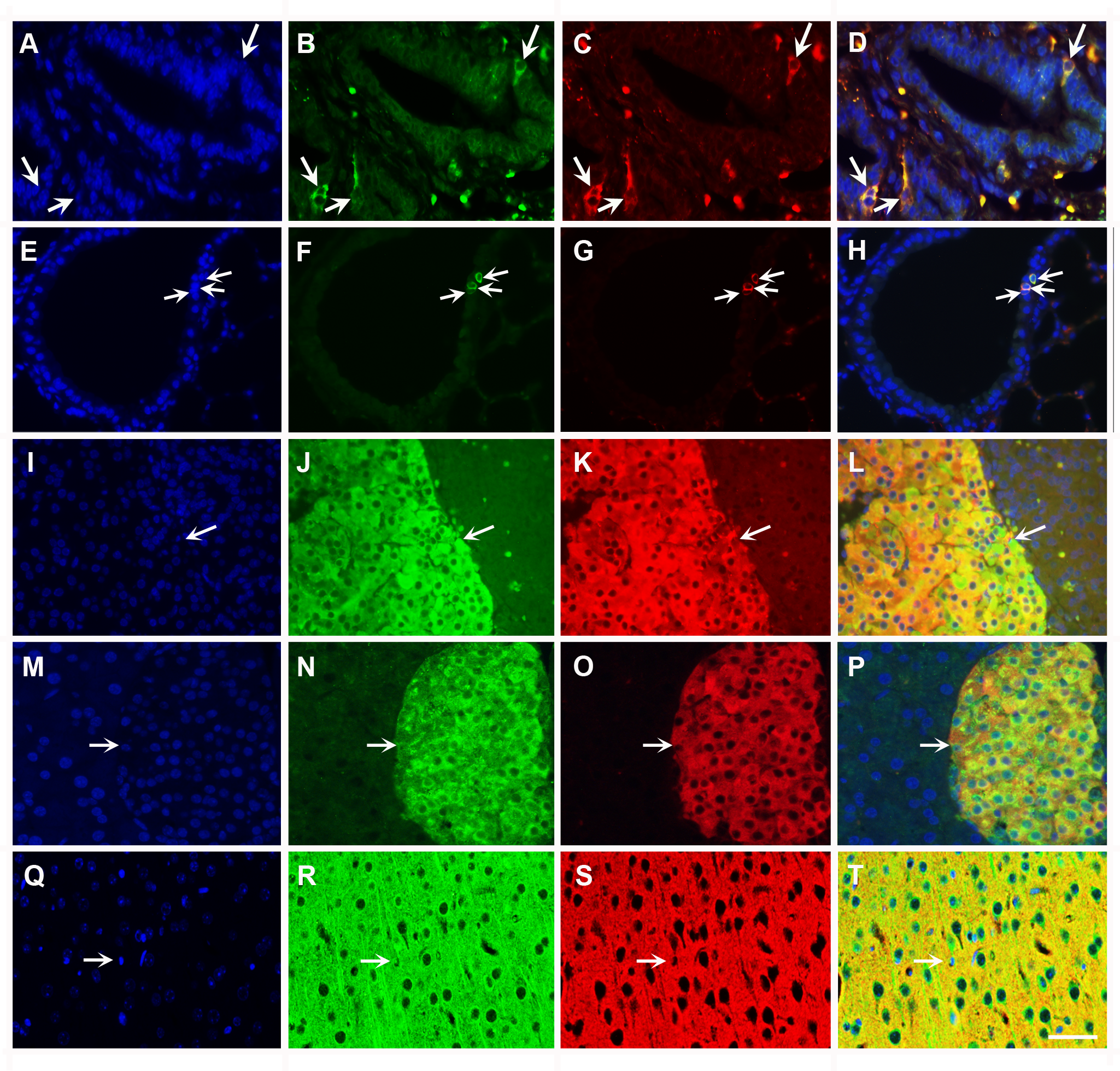

Supplement: Figure S5 — SypELDTA transgene expression has high specificity in SYP expressing cells. (A-T) Detection of EGFP (B, D, F, H, J, L, N, P, R, T, green) and SYP (C, D, G, H, K, L, O, P, S, T, red) expression (arrows) in prostate NE cells (A-D), lung NE cells (E-H), medulla of adrenal gland (I-L), islets of Langerhans in pancreas (M-P), and brain (Q-T) of SypELDTA line 147 transgenic mice. Yellow color in overlay (D, H, L, P, T) indicates co-localization of EGFP and SYP fluorescent signals. Counterstaining with DAPI (A, D, E, H, I, L, M, P, Q, T, blue). Calibration bar: 25 µm (A-D), 50 µm (E-T). (TIF) [file pone.0060905.s005.tif]

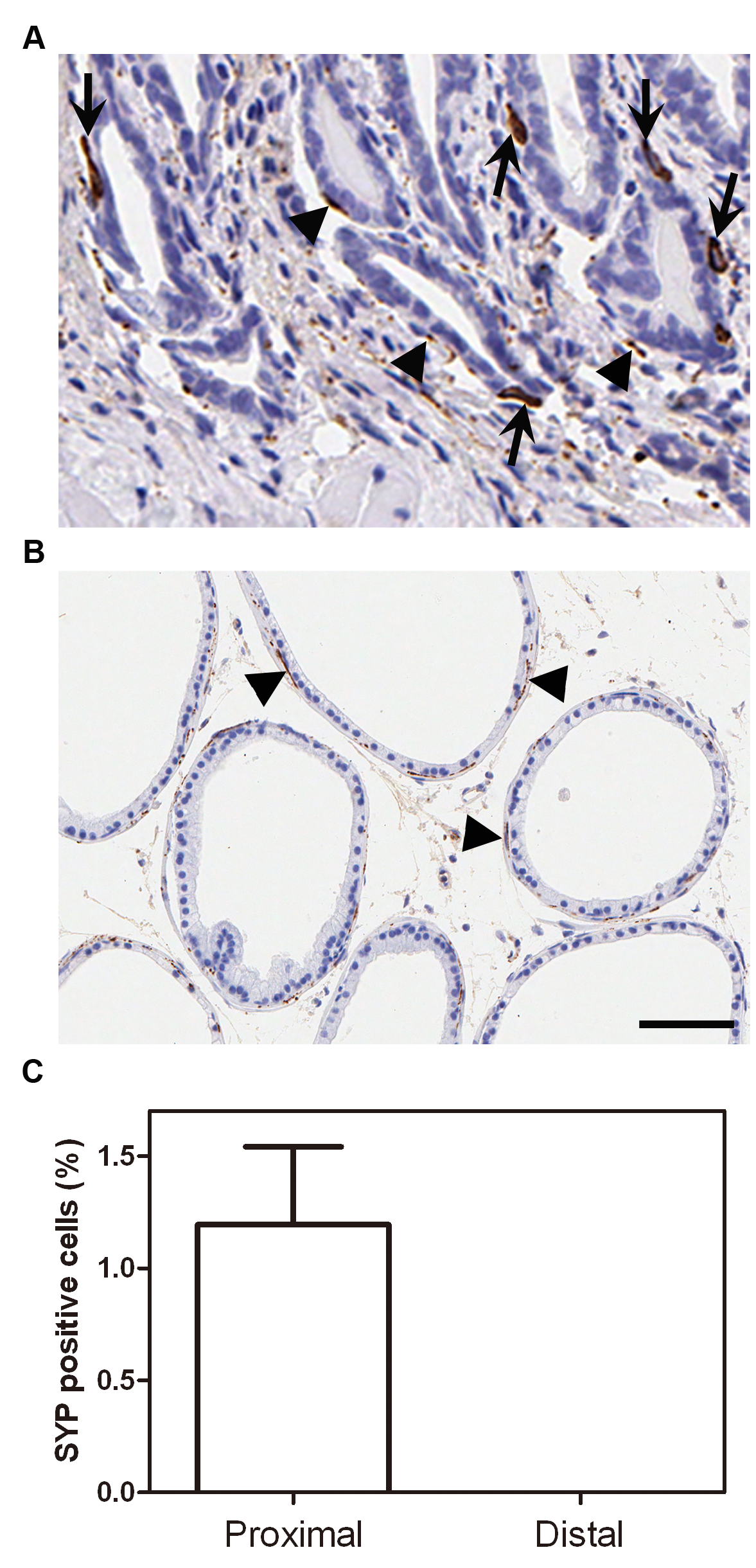

Supplement: Figure S6 — NE cells are mostly located in the proximal region of prostatic ducts. (A, B) SYP expression in NE cells in proximal (A) and distal (B) regions of prostatic ducts of the prostate (n = 6). NE cells and nerve terminals are indicated by arrows and arrowheads, respectively. Calibration bar: 50 µm (A), 100 µm (B). (C) Quantification of SYP positive NE cells. Distal regions of prostatic ducts contain no NE cells. Error bar denotes SD. (TIF) [file pone.0060905.s006.tif]

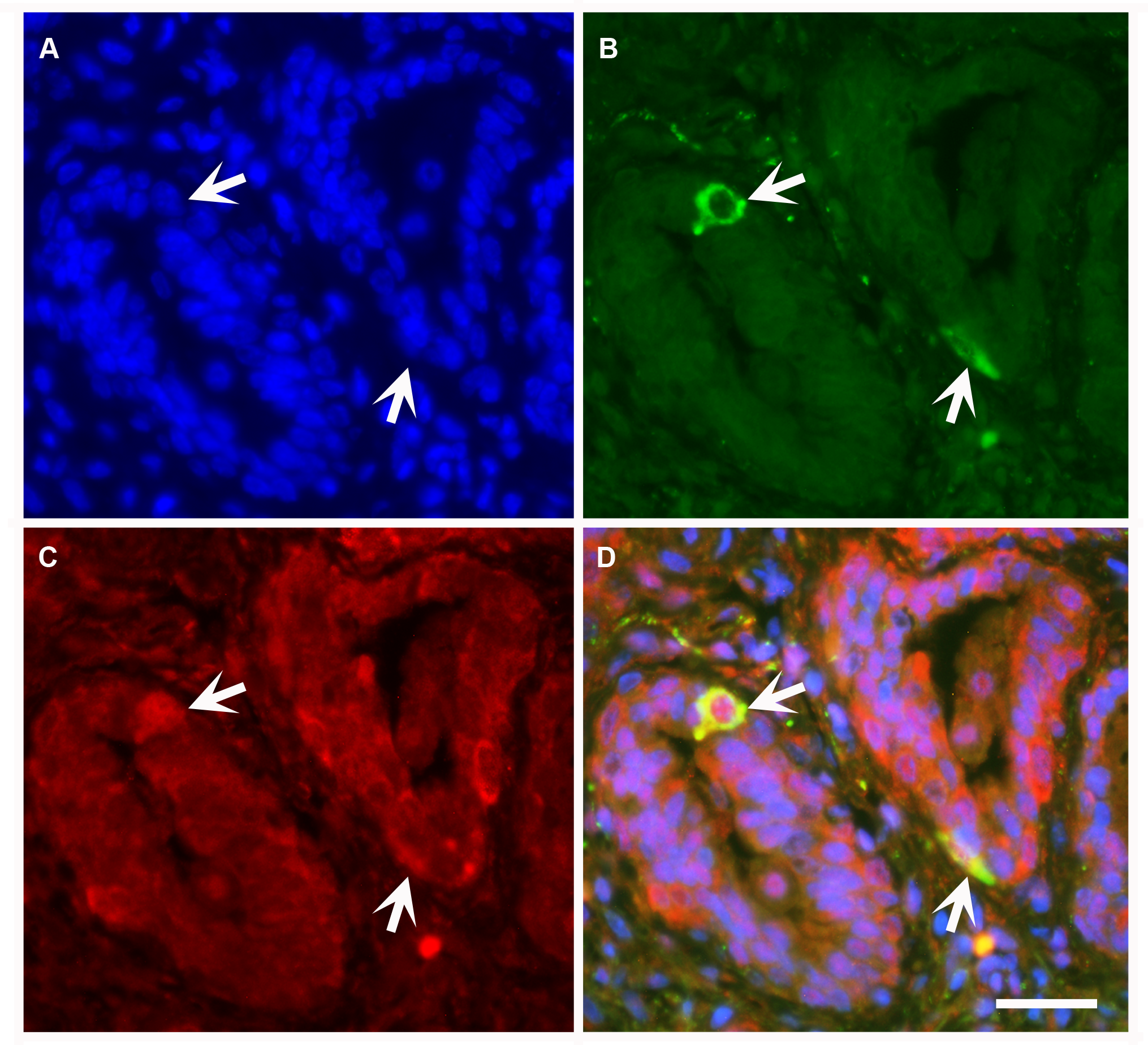

Supplement: Figure S7 — Cre recombinase under the control of probasin promoter is expressed in prostate NE cells. (A-D) Detection of SYP (B, D, green) and β-galactosidase (C, D, red, indicative of Cre-loxP mediated recombination) expression (arrows) in the prostate NE cells in PB-Cre4; R26R mice harboring Probasin-Cre and lacZ reporter gene. Yellow color in overlay (D) indicates co-localization of SYP and β-galactosidase fluorescent signals. Counterstaining with DAPI (A, D, blue). Calibration bar: 50 µm (A-D). (TIF) [file pone.0060905.s007.tif]

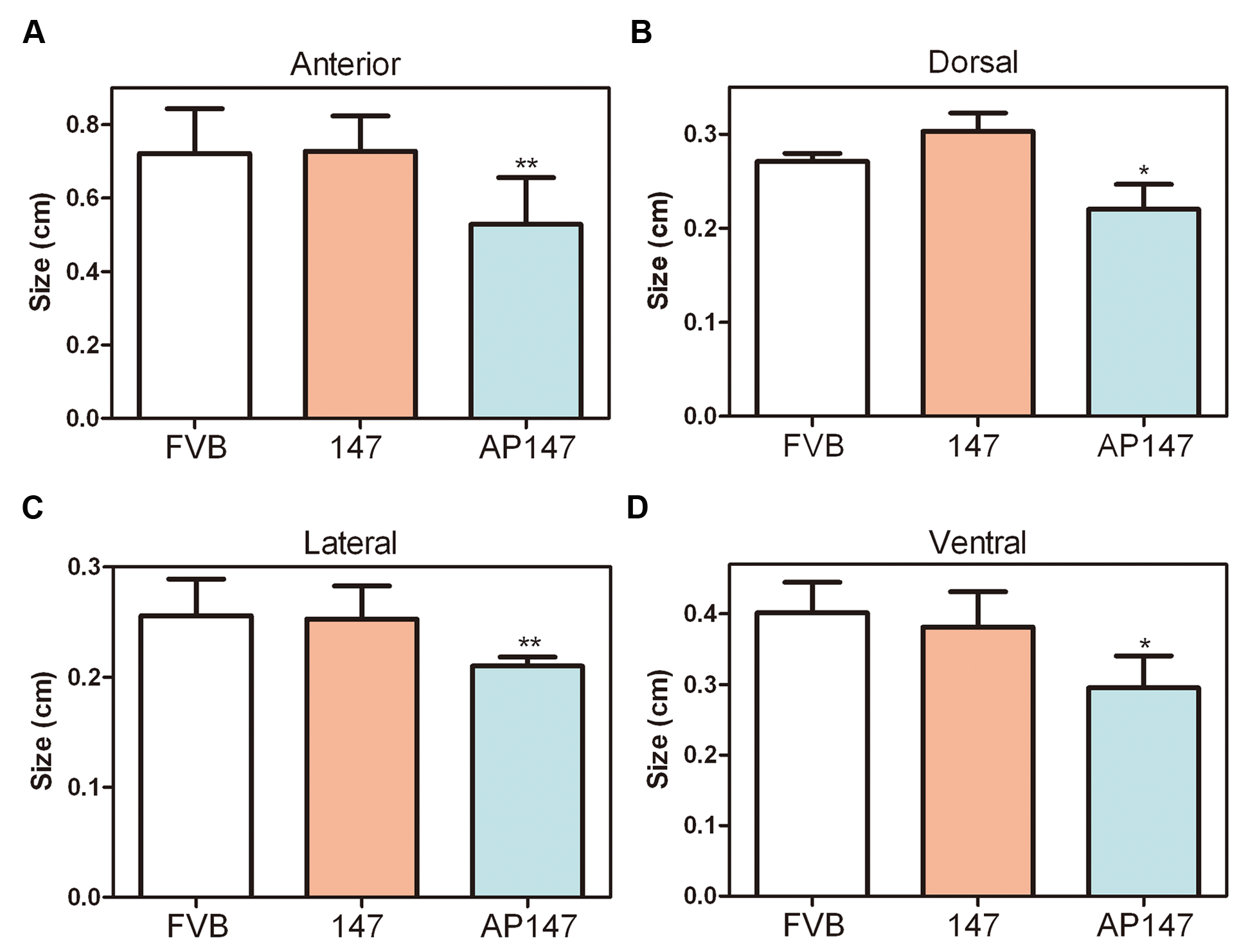

Supplement: Figure S8 — Reduced size of prostate lobes in PB-Cre4; SypELDTA mice. (A-D) Quantification of size of anterior (A), dorsal(B), lateral (C), and ventral (D) lobes among age-matched FVB/N (FVB, n = 4), SypELDTA (147; n = 4), and PB-Cre4; SypELDTA (AP147; n = 4) mice. *P<0.05. **P<0.01. Error bar denotes SD. (TIF) [file pone.0060905.s008.tif]

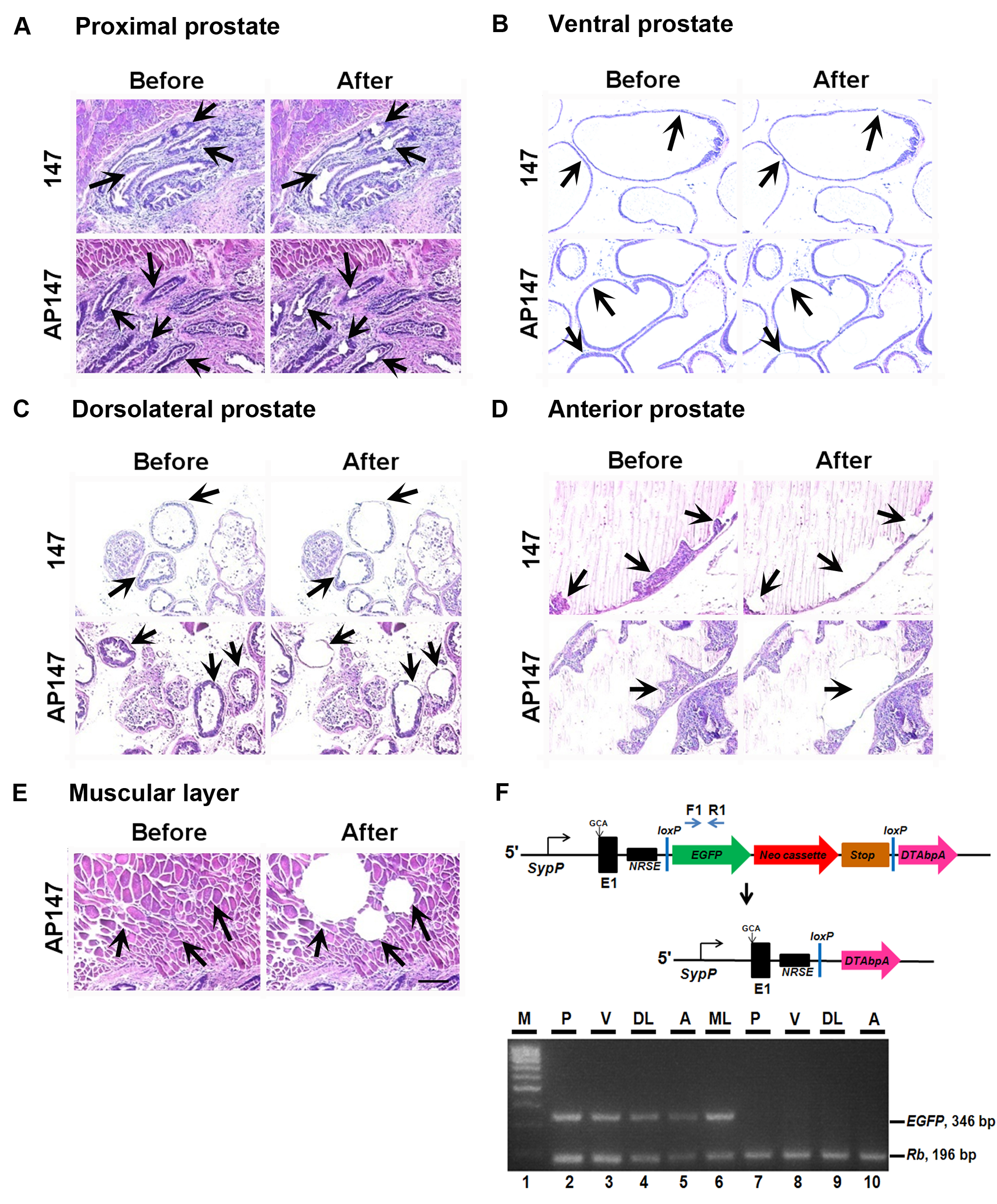

Supplement: Figure S9 — PB-Cre drives Cre- loxP recombination in the prostate of PB-Cre4; SypELDTA mice. (A-F) Microdissection-PCR. Proximal (A) and distal (ventral, B, dorsolateral, C, and anterior, D) regions of prostatic ducts and the muscular layer of the prostate (E) of age-matched SypELDTA (147) and PB-Cre4; SypELDTA (AP147) mice before and after microdissection. Hematoxylin and eosin. Calibration bar: 50 µm (A-E). (F) PCR design and detection of Cre-loxP mediated recombination in microdissected proximal region (P, lane 2 and 7), and ventral (V, lane 3 and 8), dorsolateral (DL, lane 4 and 9), anterior (A, lane 5 and 10) distal regions, and muscular layer (ML, lane 6) of prostates from SypELDTA(lanes 2-5) and PB-Cre4; SypELDTA (lanes 6-10) mice. 346 bp fragment is generated with primers F1and R1and is diagnostic for EGFP (present before Cre-loxP mediated recombination). 196 bp fragment (endogenous Rb) is internal control. Lane 1: marker (M). (TIF) [file pone.0060905.s009.tif]

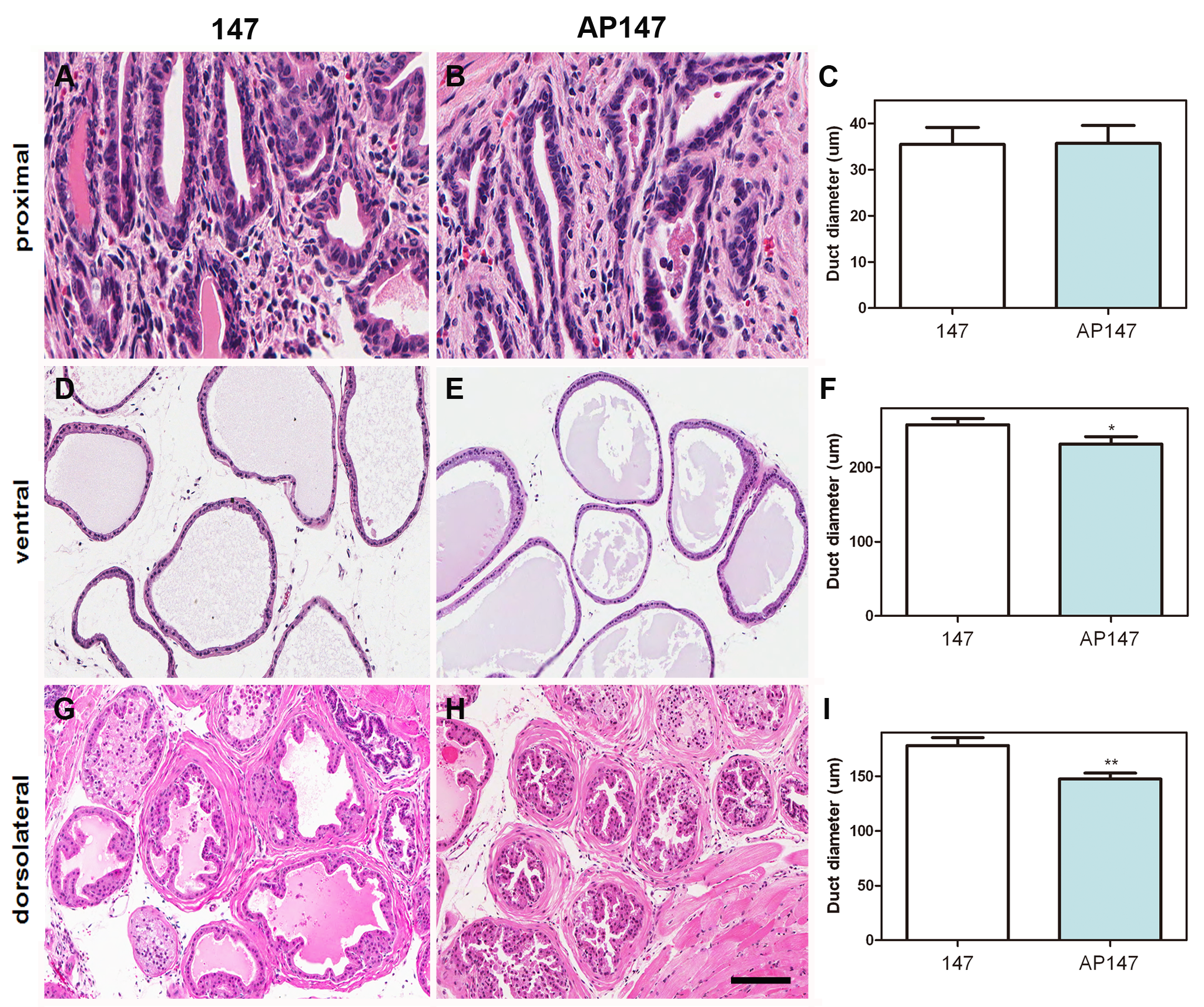

Supplement: Figure S10 — NE cell ablation results in proportional reduction of prostatic duct diameters in distal regions. (A-I) Histology (A, B, D, E, G, H) and quantification of diameters (C, F, I) of proximal (A, B, C) and distal (ventral, D, E, F, and dorsolateral, G, H, I) regions of prostatic ducts of age-matched SypELDTA (147; n = 6; A, D, G) and PB-Cre4; SypELDTA (AP147, n = 6; B, E, H) mice. Hematoxylin and eosin. Calibration bar: 50 µm (A, B), 100 µm (D, E, G, H). *P<0.05. **P<0.01. All error bars denote SD. (TIF) [file pone.0060905.s010.tif]

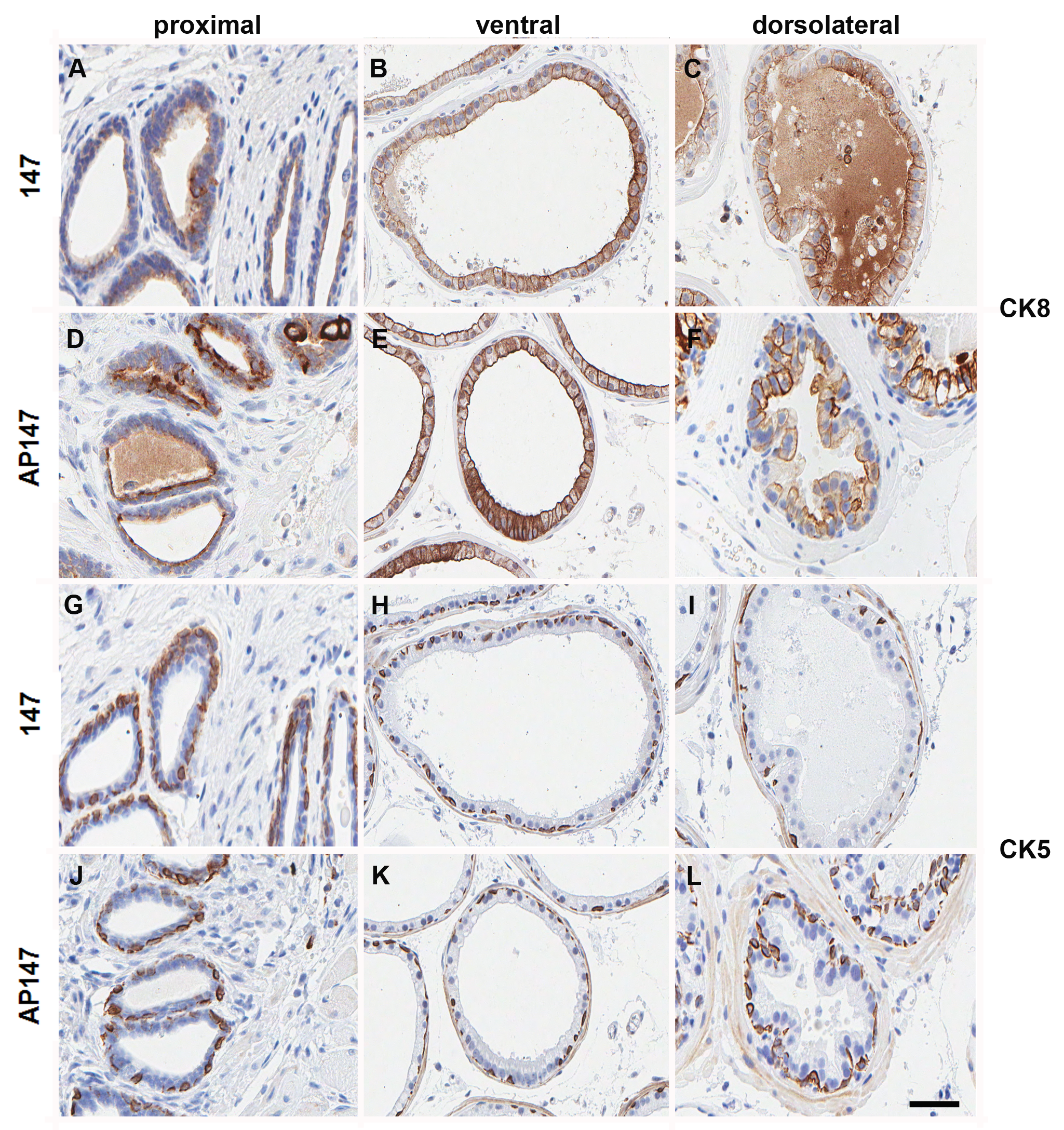

Supplement: Figure S11 — NE cell ablation does not affect luminal (CK8+) or basal (CK5+) cell differentiation. (A-L) Detection of CK8 (A-F) and CK5 (G-L) expression (brown) in epithelial cells of proximal (A, D, G, J) and distal (ventral, B, E, H, K, and dorsolateral, C, F, I, L) regions of prostatic ducts in age-matched SypELDTA (147, A-C, G-I) and PB-Cre4; SypELDTA (AP147, D-F, J-L) mice. ABC Elite method. Hematoxylin counterstaining. Calibration bar: 50 µm (A-L). (TIF) [file pone.0060905.s011.tif]

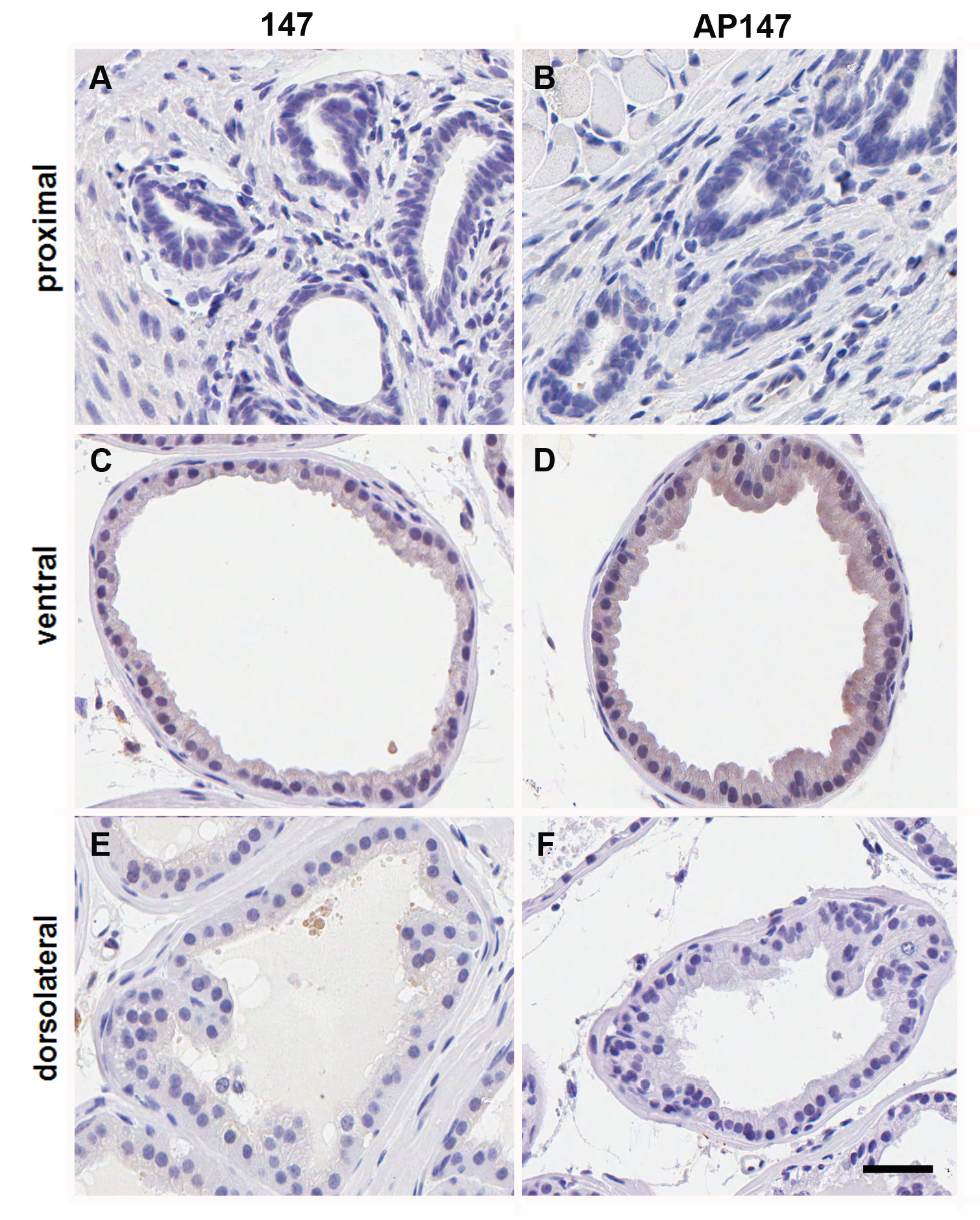

Supplement: Figure S12 — No significant cell death is observed in prostate epithelium non-NE cells in PB-Cre4; SypELDTA mice. (A-F) cleaved Caspase-3 expression in proximal (A, B), and distal (ventral, C, D, and dorsolateral, E, F) regions of prostatic ducts in age-matched SypELDTA (147, A, C, E) and PB-Cre4; SypELDTA (AP147, B, D, F) mice. The staining of embryonic dorsal root ganglia (Figure 5) served as a positive control for cleaved Caspase-3 immunostaining. ABC Elite method. Hematoxylin counterstaining. Calibration bar: 50 µm (A-F). (TIF) [file pone.0060905.s012.tif]
