# Supplementary material for: Detection and Organ-Specific Ablation of Neuroendocrine Cells by Synaptophysin Locus-Based BAC Cassette in Transgenic Mice
Source: PLoS One. 2013 Apr 22;8(4):e60905. doi: 10.1371/journal.pone.0060905 (PMC3632533; doi:10.1371/journal.pone.0060905)
Supplement: Materials and Methods S1 — Detailed protocol descriptions for generation of the targeting construct and BAC recombineering, genotyping, cell culture experiments, transgene copy number quantification, antibody sources and dilutions of immunohistochemical analyses, and microdissection-polymerase chain reaction. (DOCX) [file pone.0060905.s013.docx]

**Supporting Information - Materials and Methods**

Generation of the Targeting Construct and BAC Recombineering

The components of the BAC targeting vector were as follows: *pBS302* ([[1](#_ENREF_1)], plasmid 11925), *pcDNA3-EGFP* (D. Golenbock, plasmid 13031), and *PGKDTAbpA* ([[2](#_ENREF_2)], plasmid 13440), all from Addgene (Cambridge, MA), and *PL452* ([[3](#_ENREF_3)], National Cancer Institute - Frederick, Bethesda, MD). The ApaI-NotI DNA fragment of *loxP-EGFP-Neocassette-Stop-loxP-DTA-bpA* was made by cloning from DNA fragments of *loxP-Stop-loxP* (*pBS302*), *EGFP* (*pcDNA3-EGFP*), *Neo cassette* (*PL452*), and *DTA-bpA* (*PGKDTAbpA*), and inserted into the backbone of *PGKDTAbpA* to generate the final BAC targeting vector *pBS-loxP-EGFP-Neo cassette-Stop-loxP-DTA-bpA*. BAC clone (*RP23-267C15*), which contains *Syp* locus, was purchased from "BACPAC Resource Center" (BPRC, Oakland, CA). *Syp* homologous arms were amplified and inserted upstream and downstream of *loxP-EGFP-Neo cassette-Stop-loxP-DTA-bpA* sequence in plasmid *pBS-loxP-EGFP-Neo cassette-Stop-loxP-DTA-bpA*. 5' arm was a 510 bp fragment, containing exon 1 and *NRSE* of intron 1 within *Syp* locus, upstream of the middle of intron 1. It was generated by PCR that used forward primer (KpnI-F1) 5'-CCGTTGGGTACCTTGCTGGCACTGCTGCTGGCAGACA-3' and reverse primer (ApaI-R1) 5'-CCGTTGGGGCCCGCTCCGGGGGTGAAAGGGTCGTC-3'. To avoid possible interference of transgene expression by start codon ATG in exon 1 of *Syp* locus, we changed ATG to GCA (alanine) in forward primer of 5' arm fragment. 3' arm was a 500 bp PCR product downstream of exon 7 of *Syp* locus that was generated by the forward primer (NotI-F1) 5'-TGCCGTTGGCGGCCGCGTCCCGGCTCTTTTTCTCAGTGCGC-3', and the reverse primer (SacII-R2) 5'-CCGTTGCCGCGGAGACAGGCCTTTCATCTTGGGCGCC-3'. The homology arms were inserted after KpnI/ApaI (5' arm) and NotI/SacII (3' arm) digestion and the targeting cassette was released from the plasmid by KpnI/SacII. EL350 bacteria carrying *RP23-267C15* were electroporated with the targeting cassette DNA and selected with kanamycin and chloramphenicol. Homologous recombination was verified by sequencing of PCR products obtained using primers flanking the homology arms and *loxP-EGFP-Neo cassette-Stop-loxP-DTA-bpA* fragment. The primer sets were BAC upstream: forward primer 5'-ACTGAGCGGTCCTCTTACCACCC-3' and reverse primer 5'-CCTGCACGACGCGAGCTGC-3'; and BAC downstream: forward primer 5'-CTCCACACAGGCATAGAGTGTCTGC-3' and reverse primer 5'-CCCACTGCACCTCTGCCCAAAGA-3' (Figure S1A).

The *RP23-267C15* was cloned into *pBACe3.6* vector, which has a wild type *loxP* site. In order to eliminate potential mis-recombination between the transgene cassette and an endogenous *loxP* site in the *pBACe3.6* vector, we replaced the vector-derived *loxP* site with a *β-lactamase* sequence from *pGEM-T* vector (Promega, Madison, WI, #A3600) using homologous recombination (Figure S1B). The modified BAC construct was named *SypELDTA* (Figure 3A)*.* Furthermore, to evaluate applicability of the shorter upstream sequence of *Syp* for cell type specific expression, we used the same homologous recombination strategy and retrieved the sequence *SypP-loxP-EGFP-Neo cassette-Stop-loxP-DTA-bpA* from *SypELDTA* into *pGEM-T* vector*.* The modified BAC construct used in cell culture study was named *sSypELDTA* (Figure 3B)*.* *SypELDTA* and *sSypELDTA* constructs were purified (QIAGEN Plasmid Midi Kit, Valencia, CA, #12143) and microinjected into pronuclei of fertilized oocytes of FVB/N mice in the Cornell Transgenic Mouse Core facility.

Genotyping

*PB-Cre4* transgenic mice were identified by primers Cre5' (5'-GGACATGTTCAGGGATCGCCAGGCG-3') and Cre3' (5'-GCATAACCAGTGAAACAGCATTGCTG-3’). PCR amplification of *Cre* resulted in 296 bp DNA fragment. Endogenous *Rb* was identified by primers Rb5' (5'-CGGAAGAAGAACGTTTGTCCATTCA-3') and Rb3' (5'-CGCTGCTATACGTAGCCATTACAAC-3'). PCR amplification of *Rb* resulted in 196 bp DNA fragment. *EGFP* was identified by primers EGFP5' (5'-CCTCGTGACCACCCTGACCTACGGC-3') and EGFP3' (5'-GCCGTCCTCGATGTTGTGGCGGATC-3'). PCR amplification of *EGFP* resulted in 346 bp DNA fragment. *Neomycin* was identified by primers Neo5' (5'-GCCGCCGTGTTCCGGCTGTCAGCGC-3') and Neo3' (5'-CCGAGTACGTGCTCGCTCGATGCGA-3'). PCR amplification of *Neomycin* resulted in 342 bp DNA fragment. *DTA* was identified by primers DTA5' (5'-AGCTTGGGCTGCAGGTCGAGGGACC-3') and DTA3' (5'-ACGTCACTTTGACCACGCCTCCAGC-3'). PCR amplification of *DTA* resulted in 348 bp DNA fragment. The PCR temperature profile was 35 cycles for 94°C for 30 seconds, 60°C for 1 minute, and 72°C for 2 minutes with extension of the last cycle for 10 minutes at 72°C [[4](#_ENREF_4)].

Cell Culture Experiments

Mouse *p53* and *Rb* double deficient prostate adenocarcinoma cell lines PCN1 and PCN3 were established from prostate carcinomas of *PB-Cre4; p53^loxP/loxP^Rb^loxP/loxP^* mice [[5](#_ENREF_5)]. They were maintained in the Dulbecco's Modification of Eagle's Medium (DMEM, Cellgrow, Manassas, VA, #15-018-CV), supplementary with fetal bovine serum (FBS, Invitrogen, Carlsbad, CA, #16000-044), L-Glutamine (Cellgrow, #25-005-Cl), Na-Pyruvate (Cellgrow, #25-000-Cl), and Penicillin-Streptomycin (Cellgrow, #30-002-Cl).

For EGFP detection experiment, PCN1 and PCN3 cells were seeded in triplicate in 12-well plates and transfected the next day with *sSypELDTA* using Lipofectamine 2000 (Invitrogen, #11668019). 48 hours after transfection, EGFP signal was observed via fluorescent microscopy.

For DTA detection, cells were transfected with plasmids *PGKDTAbpA*, *sSypELDTA*, and *SypDTA*. *sSypELDTA* transfected cells were infected by Adenovirus-*Cre* (Ad-*Cre*) 48 hours post-transfection. 24 hours after infection, RT-PCR was carried out for all groups. RNA was isolated using mirVana miRNA Isolation Kit (Ambion, Austin, TX, #AM1561) according to the manufacturer's protocol. RNA concentration and purity were determined by NanoDrop. cDNA was prepared from 100 ng total RNA using SuperScript III (Invitrogen, Carlsbad, CA, #18080-051) and amplified with DTA forward primer 5'-ATGGATCCTGATGATGTTGTTGATTCTTCTAAATC-3' and a reverse primer 5'-TTAGAGCTTTAAATCTCTGTAGGTAGTTTGTCCAA-3', yielding a 657 bp PCR product. The PCR temperature profile was 30 cycles for 95ºC for 45 seconds, 60ºC for 45 seconds, and 72ºC for 1 minute with the extension of last cycle for 1 minute at 72ºC.

For detection of Cre-*loxP* mediated recombination, EL350 cells were cultured in 10 ml of LB broth at 32°C overnight. 10 ml EL350 was divided into two 5 ml EL350 vials. One EL350 vial was treated with 100 μl 10% arabinose and other EL350 vial served as a control without arabinose induction. After one hour culture at 32°C, EL350 cells were electroporated with *sSypELDTA* and selected with ampicillin on LB plate. After 16 hours incubation at 32°C, colonies were picked, and Cre-*loxP* mediated recombination was verified by sequencing of PCR products obtained using forward primer (F1) 5'-CTCACTGCCGCAGAGGGGGCCTCCA-3', forward primer (F2) 5'-ATGGATCCTGATGATGTTGTTGATTCTTCTAAATC-3' and a reverse primer (R1) 5'-ACGTCACTTTGACCACGCCTCCAGC-3'. Combination of F1 and R1 primers and that of F2 and R1 yielded 617 bp and 256 bp PCR products, respectively.

Transgene Copy Number Quantification

Transgene copy number was carried out by Southern blotting and qPCR. Genomic DNA was isolated with a Gentra Puregene Tissue Kit (Qiagen, #158667). Genomic DNA from the *sSypELDTA* mouse (line 141) was digested by restriction enzymes AflII, AscI, and PacI (New England Biolabs, Ipswich, MA), and detected by Southern blotting with ^32^P-labeled *EGFP* probe. 1117 bp *EGFP* probe was prepared by PCR using primers 5'EGFP (5'-ATGGTGAGCAAGGGCGAGGAGC-3') and 3'EGFP (5'-CCTGCACGACGCGAGCTGC-3'). The copy number of *sSypELDTA* transgene was estimated by densitometric analysis with Image J software (NIH, Bethesda, MD). To perform qPCR, 60 ng DNA was amplified with custom TaqMan real time *EGFP* probe (forward primer: 5'-CACATGAAGCAGCACGACTT-3'; reverse primer: 5'-GTGCGCTCCTGGACGTA-3'), followed by normalization to the endogenous *β-actin*. All PCR reactions were performed in triplicate on AB 7500 Real Time PCR system (Applied Biosystems Inc, Foster City, CA). After getting relative quantification among each line, copy number was estimated by normalizing to copy number of *sSypELDTA* transgene in line 141*.*

Immunohistochemical Analyses - Antibody Sources and Dilutions

Primary antibody: rat anti-CK8 (Develop-mental Studies Hybridoma Bank, University of Iowa, IA, #TROMA-I, 1:10), rabbit anti-CK5 (Covance, NJ, # PRB-160P, 1:1000), mouse anti-SYP (BD Biosciences, San Jose, CA, #611880, 1:200), rabbit anti-cleaved Caspase-3 (Cell Signaling, Danvers, MA, #9661, 1:200), rabbit anti-GFP (NOVUS biological, Littleton, CO, #NB600-303, 1:1000), rabbit anti-β-galactosidase (abcam, Cambridge, MA, #ab616-1, 1:200). Secondary antibody: biotinylated goat anti-mouse (Vector Laboratories, Burlingame, CA, # BA-9200, 1:200), biotinylated goat anti-rabbit (Vector Laboratories, #BA-1000, 1:200), biotinylated goat anti-rat (Vector Laboratories, # BA-9400, 1:200), donkey Alexa-Fluor 594-conjugated anti-mouse (Invitrogen, Carlsbad, CA, #A21203, 1:200), donkey Alexa-Fluor 488-conjugated anti-rabbit (Invitrogen, #A21206, 1:200), donkey Alexa-Fluor 594-conjugated anti-rabbit (Invitrogen, #404239, 1:200), donkey Alexa-Fluor 488-conjugated anti-mouse (Invitrogen, #A21202, 1:200).

Microdissection- Polymerase Chain Reaction

For laser microdissection, 4-μm-thick paraffin sections were prepared on PET (polyethylene terephthalate)-membrane slides for laser microdissection (Leica microsystems, Buffalo Grove, IL, #11505151) and stained with hematoxylin and eosin. Prostate cells from different areas (proximal region, ventral lobe, dorsolateral lobe, anterior lobe, muscular layer) were microdissected using a blue laser (Laser Microdissection System, Leica AS, Heidelberg, Germany), collected into caps of 0.6 ml Eppendorf tubes filled with lysis buffer, digested in proteinase K, divided equally among tubes for detection of recombination and used for subsequent PCR amplification following previously described protocol [[4](#_ENREF_4)]. *EGFP* was identified by primers EGFP5' (5'-CCTCGTGACCACCCTGACCTACGGC-3') and EGFP3’ (5'-GCCGTCCTCGATGTTGTGGCGGATC-3'). PCR amplification of *EGFP* resulted in 346 bp DNA fragment.

**References**

1. Sauer B (1993) Manipulation of transgenes by site-specific recombination: use of Cre recombinase. Methods Enzymol 225: 890-900.

2. Soriano P (1997) The PDGF alpha receptor is required for neural crest cell development and for normal patterning of the somites. Development 124: 2691-2700.

3. Liu P, Jenkins NA, Copeland NG (2003) A highly efficient recombineering-based method for generating conditional knockout mutations. Genome Res 13: 476-484.

4. Nikitin A, Lee WH (1996) Early loss of the retinoblastoma gene is associated with impaired growth inhibitory innervation during melanotroph carcinogenesis in Rb+/- mice. Genes Dev 10: 1870-1879.

5. Zhou Z, Flesken-Nikitin A, Corney DC, Wang W, Goodrich DW, et al. (2006) Synergy of p53 and Rb deficiency in a conditional mouse model for metastatic prostate cancer. Cancer Res 66: 7889-7898.
